# Supplementary material for: Environmental connectivity controls diversity in soil microbial communities
Source: Commun Biol. 2021 Apr 22;4:492. doi: 10.1038/s42003-021-02023-2 (PMC8062517; doi:10.1038/s42003-021-02023-2)
Supplement: Supplementary file 2 — Supplementary Information [file 42003_2021_2023_MOESM2_ESM.pdf]

Supplementary information to

Environmental Connectivity Controls Diversity in Soil Microbial Communities

Manupriyam Dubey[1], Noushin Hadadi[1], Serge Pelet[1], Nicolas Carraro[1], David R. Johnson[2], and Jan R. van der Meer[1]*

[1] Department of Fundamental Microbiology, University of Lausanne, 1015 Lausanne, Switzerland
[2] Department of Environmental Microbiology, Swiss Federal Institute of Aquatic Science and Technology, Eawag, CH 8600 Dübendorf, Switzerland

Table of contents **Page**

Supplementary figure 1 3
Supplementary figure 2 4
Supplementary figure 3 5
Supplementary figure 4 6
Supplementary figure 5 7
Supplementary table 1 : 8
Supplementary table 2 9
Supplementary Note 1 11
Supplementary methods 12
Section 1 Community modeling general terms 12
1.1 General parameters 12
1.2 Build the time vector and the starting community structure. 12
Section 2: Simulation of growth in high connectivity conditions 13
2.1 Random distribution of growth rates among starting OTUs 13
2.2 Attribution of growth rates randomly proportionally to the OTU distribution 14
2.3 Simulation of cell death 14
2.4 Simulation of liquid growth 15
Section 3. Low connectivity growth 16
3.1 Attributing growth rates to cells in beads 17
3.2 Attributing interaction effects to OTU pairs in beads 18
3.3 Initializing the bead growth simulation in the null model (no interactions). 18
3.4 Imposing different interaction effects to both partner in every bead 23
3.4.1 Bimodal interaction coefficients 24
3.4.2 Biased random positive and negative interactions 25
3.4.3 Random positive influence on slow growers 26
3.4.4 Random interaction 27
3.4.5 Biased growth penalty on slow growers 28
Section 4. Preparation of agarose beads with encapsulated cells. 30
Supplementary reference 35

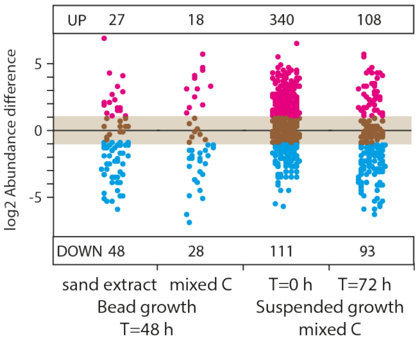

Supplementary figure 1.

In- and decrease of the number of OTUs under low or high connectivity growth in comparison to SC cells at start. Jitter plots illustrate the $\log_2$ normalized abundance difference of individual OTUs for low (i.e., bead growth, sampled at t = 48 h incubation time, for two substrates *sand extract* and *mixed C*) or high (i.e., suspended growth, taken at start of incubation and after 72 h in *mixed C* medium) connectivity environments. UP, number of OTUs increasing abundance more than twofold (magenta-colored); DOWN, decreasing abundance more than twofold (cyan-colored). Absent OTUs compared to the SC starting suspension not counted.

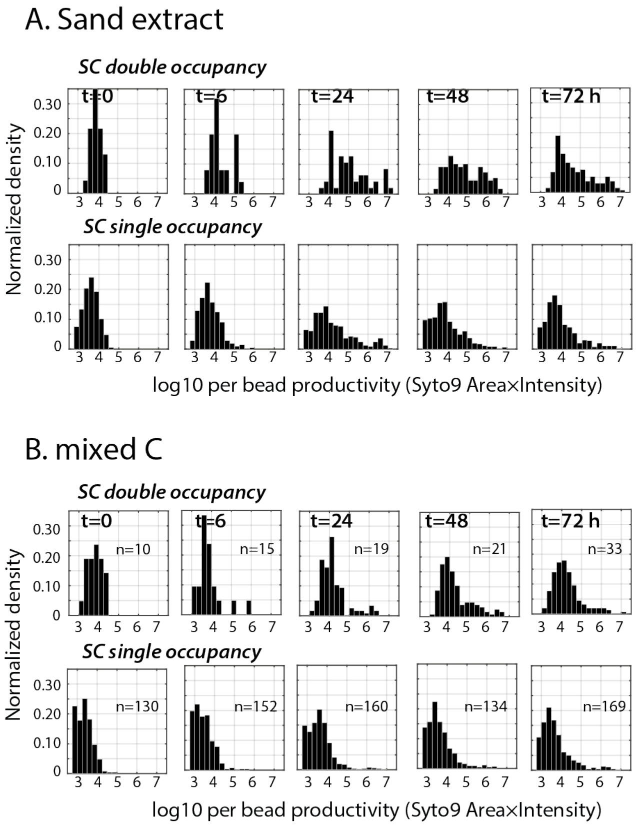

Supplementary figure 2.

Improved productivity of sand community founder cell pairs as opposed to single starting cells in low connectivity environments. Normalized distributions of log_{10} per bead productivities of microcolony growth (measured as SYTO9 fluorescence intensity times particle area) for SC cells on sand extract (a) or mixed C substrates (b) at different sampling time points, as indicated. SC double occupancy, beads showing 2 or more microcolonies. SC single occupancy, beads with only 1 microcolony. *n* = number of beads in that category and time point.

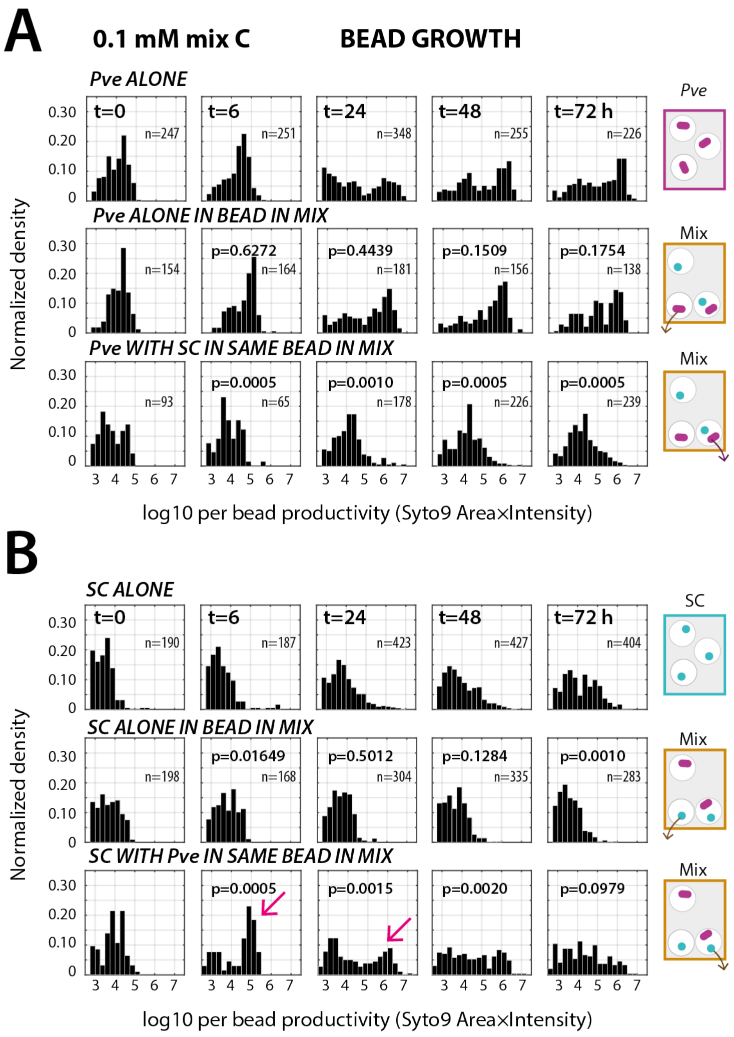

Supplementary figure 3.

Improved growth of sand community cells randomly paired with a pure culture soil bacterium.
a Normalized per bead producitivities of the *P. veronii* partner organism to SC cells (as log10 SYTO9 fluorescence intensity times particle areas) taken at different sampling time points, as indiciated, using mixed C substrates (0.1 mM C in total). *Pve alone*, incubation of encapsulated *P. veronii* by itself. *Pve alone in bead in mix*, beads that perchance carry only *P. veronii* colonies but that were present in the mixture incubation with SC. *Pve with SC in same bead in mix*, signal of *P. veronii* microcolonies in beads that also carried SC microcolonies. **b** as for **a**, but for the SC partners. *SC* alone, incubation of encapsulated SC cells by themselves. *SC alone in bead in mix*, beads that perchance carry only *SC* colonies but that were present in the mixture incubation with *P. veronii*. *SC with Pve in same bead in mix,* signal of *SC* microcolonies in beads that also carried *P. veronii* microcolonies. p-values derive from Fisher's comparison of distributions to that of the incubation (SC or *Pve*) alone. Distributions were pooled from independent biological triplicates. *n* notes the number of observed beads for that category. Note how *SC* community growth (as $\log_{10}$ PBP) is statistically significantly better for beads that have *P. veronii* as a partner.

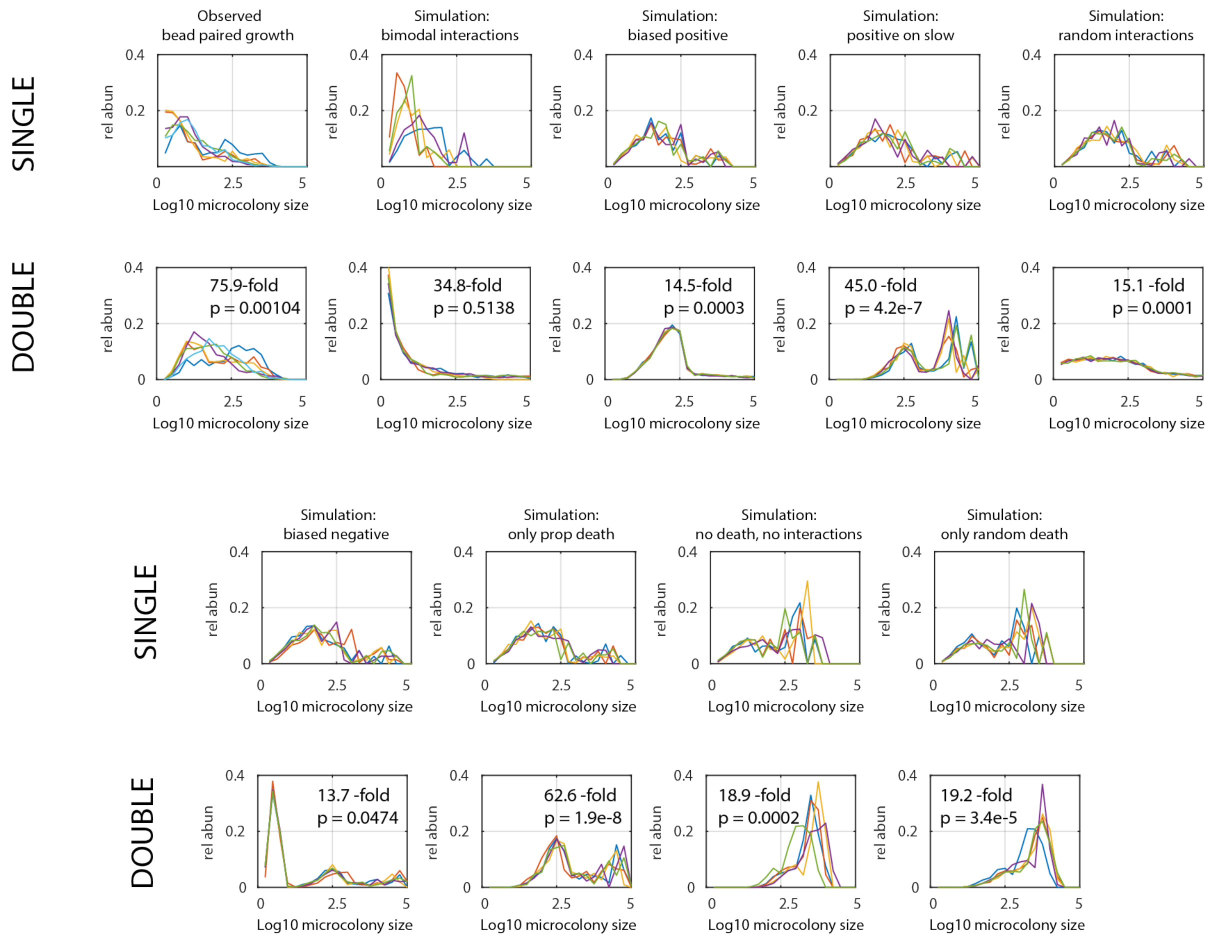

Supplementary figure 4.

Simulated growth of paired versus single founder cells in low connectivity.
Plots show log10-transformed probability normalized distributions of five independent simulations of steady-state microcolony sizes for beads with a single starting (SINGLE) or paired founder (DOUBLE) cell. Simulation scenarios show the different imposed global interspecific interactions and/or cell death at start. All simulations start with 75% of beads with single and 25% with paired founder cells, and a total of 200,000 beads. Distribution curves result from subsampling of 5000 *in silico* beads from the total pool at the last time step. Rel abun, relative abundance. Fold-values are the mean ratio of the 75th percentiles of the double versus single microcolony size distribution, with p-values corresponding to the probability of the *null* model being correct (i.e., no difference among 75th percentiles in single and double, n = 5 simulations).

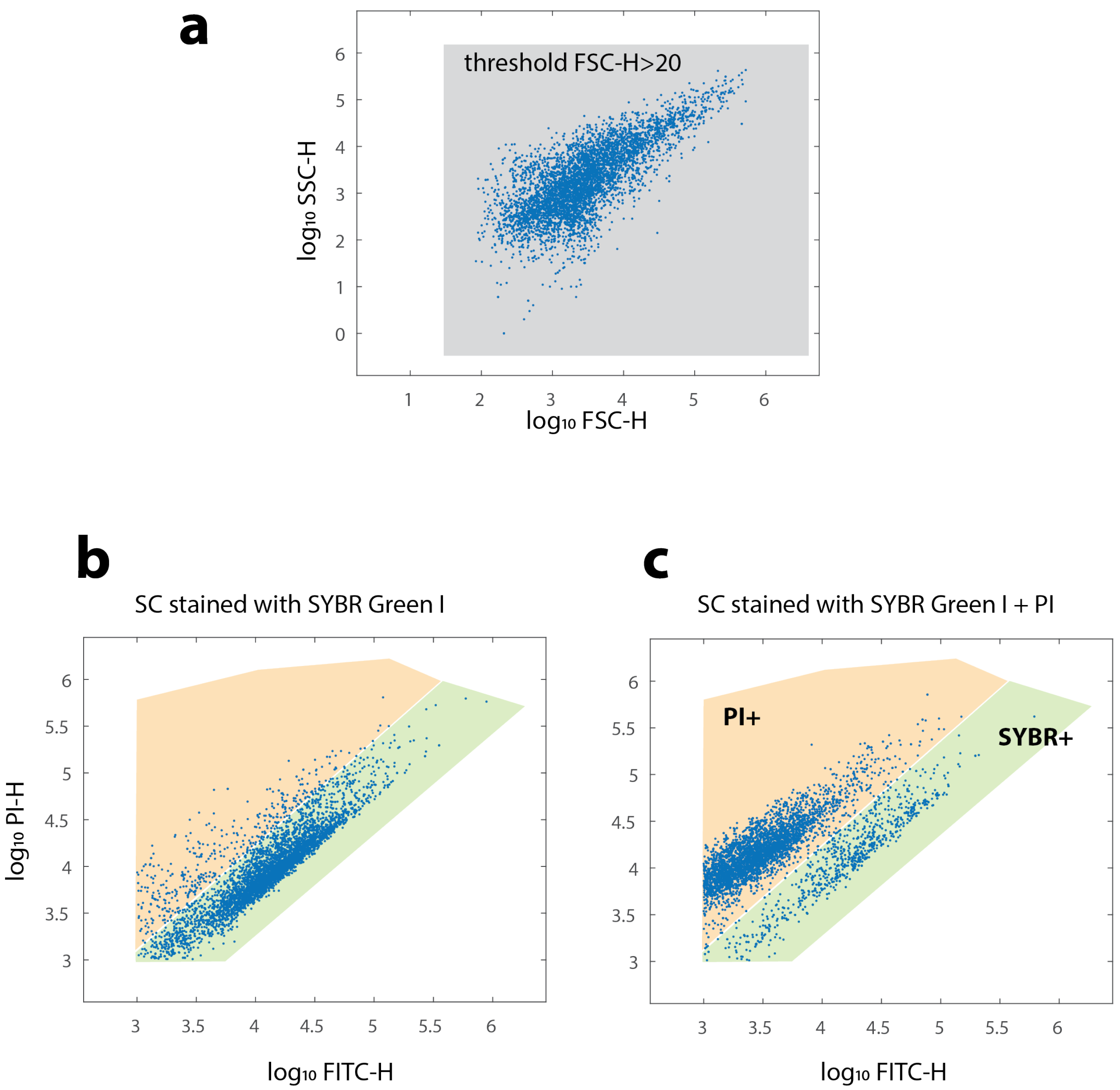

Supplementary figure 5.

Gating strategy for flow cytometry.
a. Stained soil community cell populations are filtered at FSC-H levels above 20. On the basis of samples stained with either SYBR Green I alone (**b**), or SYBR Green I and Propidium iodide (PI, **c**), we define the PI+ (light orange background) and SYBR+ (light green background) gates. Only values above PI and SYBR Green I fluorescence thresholds of 1000 are considered. Note that SYBR Green I is measured within the FITC-H channel of the instrument.

Supplementary table 1 :
Mean number of microcolonies per bead in the various incubations

Substrate	time point	Mean number of SC microcolonies per bead (± one *sd*)[a]
mixed C (exp 071117)	T0	1.11 (±0.35)
	T6	1.14 (±0.38)
	T24	1.52 (±0.92)
	T48	1.60 (±1.19)
	T72	2.29 (±2.26)
mixed C (exp 250817)	T0	1.28 (±0.59)
	T6	1.28 (±0.57)
	T24	2.88 (±0.85)
	T48	1.54 (±0.99)
	T72	1.61 (±1.00)
sand extract	T0	1.24 (±0.52)
	T6	1.27 (±0.57)
	T24	1.62 (±1.52)
	T48	2.19 (±1.92)
	T72	2.73 (±3.83)

a) *sd,* standard deviation, calculated from biological triplicates.

Supplementary table 2
Isolated DNA amounts from low and high connectivity growth

Subtrate	cells	connectivity	amount (ng)	amount of beads	amount of cells
sand extract	SC+PVE	low	10.71	2.4*10e6	
sand extract	SC+PVE	low	8.75	2.4*10e6	
sand extract	SC+PVE	low	10.01	2.4*10e6	
sand extract	SC	low	9.24	2.4*10e6	
sand extract	SC	low	16.24	2.4*10e6	
sand extract	SC	low	6.02	2.4*10e6	
mixC	SC	low	31.92	2.4*10e6	
mixC	SC	low	14.49	2.4*10e6	
mixC	SC	low	32.48	2.4*10e6	
mixC	SC	high	11.62		1.5*10e8
mixC	SC	high	17.92		1.5*10e8
mixC	SC	high	14.07		1.5*10e8
mixC	SC	high	18.69		1.5*10e8

	paired T-test, two-sided
H_0: DNA from mixC in high = DNA from mixC in low	0.09983964
H_0: DNA from mixC in high = DNA from SC in low	0.037223924

We further assume from microcolony size distributions that we would have on average 100 cells per bead, which gives approximately the same number of cells in the isolation procedure.

Supplementary Note 1

Microcolony size calculation.

For all results we estimated microcolony growth as the product of the increased imaged fluorescence area of ‘objects’ inside beads and the mean pixel intensity across the object. Since we cannot measure true biovolumes of microcolonies inside individual beads, the imaged fluorescence area (as a two-dimensional cross-section of the microcolony) and pixel intensity (a proxy for the height of the microcolony) was utilized. The reasoning for using fluorescence intensity was that multiple cell layers will proportionally increase measured pixel intensity for as long as the camera signal is not saturated, and for as long as microcolonies do not surpass 3-5 cell layers.
As an example, single cells of *P. veronii* imaged at start showed an area of 25 pixels and a mean SYTO9 pixel intensity of 800, equalling a product of 2×10^4 (pixels×intensity). At time 24 h, imaged objects showed areas of 500 pixels and mean pixel intensity of 1500 (=7.5×10^5 pixels×intensity). Such object would then be considered to contain 38 cells (7.5×10^5 divided by 2×10^4).

To estimate the maximum number of cell divisions in beads, we compared the ranges of maximum microcolony areas in individual beads after 24–72 h of growth compared to that at time of inoculation. Observed maximum increases in histogram plots of microcolony area × intensity (e.g., Fig. S1 and S2), correspond to a factor of 1000-fold ($\log_{10}$ = 3.25 is single cell cut-off at start, maximum $\log_{10}$ PBP at t=72 is ~6.5–7). This would thus correspond to a logaritmic growth of 2^{10} = 1024 from a single starting cell in a bead (=10 generations).

Also solely based on area of imaged microcolonies, the maximum imaged area increase amounted to some 50–100-fold within 24 h. Assuming a round microcolony with densely packed cells, this would correspond to approximately 450–1000 cells from a single starting cell, which is equivalent to 9–10 generations. Of note, therefore, that we limited the external substrate concentration to 0.1 mM (mixed-C) to avoid overgrowth of microcolonies inside the beads, which would lead to cell escape and their subsequent proliferation outside the beads.

Supplementary methods

Section 1 Community modeling general terms

Simulation of microbial community diversity and composition in (i) liquid suspension (high connectivity environment) and (ii/iii) where species are encapsulated in beads, for either a single cell or pairs of cells (low connectivity environment).

We compare the final steady state OTU diversity (distributions, paired- or single microcolony growth and diversity measures) in all three scenarios to assess the effect of connectivity.
Below we give the specific code implementation of the simulations in MatLab (v. 2016a). The general terms and concepts of the simulations are presented in the main Method part.

Full code examples can be retrieved [1].

1.1 General parameters

We use the exact same initial conditions for all scenarios, starting with 200,000 cells (corresponding to the $2 \cdot 10^5$ cells per ml in the experiments at start). We simulate 120 time steps, corresponding to 60 h. All growth kinetic parameters except growth rate are kept the same for all species. The initial carbon concentration corresponds to the experimental values, allowing similar community development in terms of its size (i.e., cell numbers).

```
%1.1.1 Common parameters for all experiments

No_cell_community_start=2.00E+05;% ml-1
deltaT = 2;
experiment_Length = 120; % in hours (gen time is between 1h to 24 h )
initial_Carbon_concentration = 0.05E-3; % in g/ml. This is the initial carbon concentration
equally available for all cells
Smin = 3E-6; % in g/ml. This is the minimum concentration at which growth stops.
Mu_max=0.6; % maximally imposed Mu for all cells under all conditions.
biomass_per_cell = 1.20E-13; % in gr C. Equal for all species.
Ks = 0.3E-6; % in g/ml; equal for all species.
yield = 0.3; % carbon to biomass yield in g/g. Equal for all species.
```

1.2 Build the time vector and the starting community structure.

We take the species composition from the experimentally determined distribution of 16S rRNA amplicon sequencing in the washed soil suspensions before inoculation (available as 'SC_clean.mat'). This is transformed to an OTU structure table, which is then used to calculate the probability of sampling an OTU at t=0 in the simulations.

```
%1.2.1

Time = 0:deltaT:experiment_Length;

%% We need the following .mat file that contains the log10 read values for each OTU.

load SC_clean.mat


% Extract the log abundance values of this file and produce a matrix with the first column
being the species number and the second the corresponding log abundances

OTU_Value = power(10,SC_clean);
OTU_Value(:,6) = (1:length(OTU_Value));
OTU_Value = OTU_Value(:, [6 1]);
keep = OTU_Value(OTU_Value(:,2)>0);
OTU_Value = OTU_Value(keep,:);

% Negative and NaN values are removed. The index of each OTU in the initial file is kept to
follow their changes afterward.
```

```
No_OTUs= length (OTU_Value);
```

Next we build a "community_growth" data structure that for each OTU in the list will collect the information on their Mu, Ks, yield and potential species interactions.

```
%1.2.2 Community_growth data structure

community_growth.OTU = OTU_Value(:,1);
community_growth.OTU_Value = OTU_Value(:,2);
```

The starting number of cells for each OTU is sampled from the probability of occurrence of each OTU.

```
% 1.2.3 Build the probability_vector, which guides the sampling of different OTUs the 2E5
initial cells.

probability_vector=100*(OTU_Value(:,2)/(sum(OTU_Value(:,2))));

OTU_id_per_Cell =
datasample(community_growth.OTU,No_cell_community_start,'Weights',probability_vector);
[cell_distribution_per_OTU,a] = histcounts(OTU_id_per_Cell,OTU_Value(:,1));
cell_distribution_per_OTU=[cell_distribution_per_OTU 0];

% The cell_distribution_per_OTU now gives the number of cells per OTU across 2E5 total cells
at start.
```

Section 2: Simulation of growth in high connectivity conditions

First, we are contrasting simulations of growth in high connectivity conditions using two different distributions of growth rates (which are *a priori* unknown). In the first case, we chose OTU growth rates randomly; in the second, we attribute growth rates to OTUs according to their $\log_{10}$ probability of relative abundance. Steady-state community outcomes are compared to experimental data from liquid-grown SC cultures in order to determine which scenario corresponds better. This scenario of growth rate attributions is then kept in subsequent simulations of low connectivity conditions.

2.1 Random distribution of growth rates among starting OTUs

In the first case, the inherent maximum growth rates are attributed randomly among the OTUs. The ‘absolute’ µmax is set to 0.6 and can vary between 0.01 and 0.6. The following loop adds a list of growth rates to each of the OTUs in the community_growth structure.

```
%2.1.1 Random distribution of growth rates up to a maximum

for i=1:No_OTUs
   community_growth.Mu(i,1)=(0.01+0.6.*rand(1));
    if community_growth.Mu(i,1) > Mu_max
        community_growth.Mu(i,1) = Mu_max.*rand(1);
    end
end
```

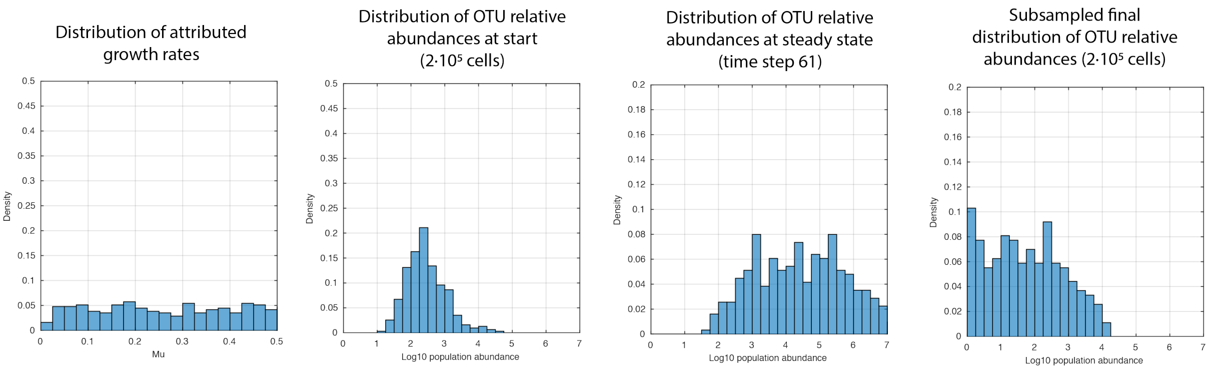

2.2 Attribution of growth rates randomly proportionally to the OTU distribution

In the second case, the inherent maximum growth rates are attributed by weighing the original log_{10} OTU distribution plus adding a randomized effect. The argument here is that the OTU distribution of cells as we wash them from soil must be to some extent a reflection of their growth properties, including growth rate, with more abundant taxa being on average faster growers.

```
%2.2.1 OTU proportional attribution of growth rates
B=log10(OTU_Value(:,2));
prob_vector=100*(B/sum(B));

for i=1:No_OTUs
   community_growth.Mu(i,1)=prob_vector(i).*(0.1.*rand(1)+0.6);
    if community_growth.Mu(i,1) > Mu_max
        community_growth.Mu(i,1) = Mu_max.*rand(1);
    end
end
```

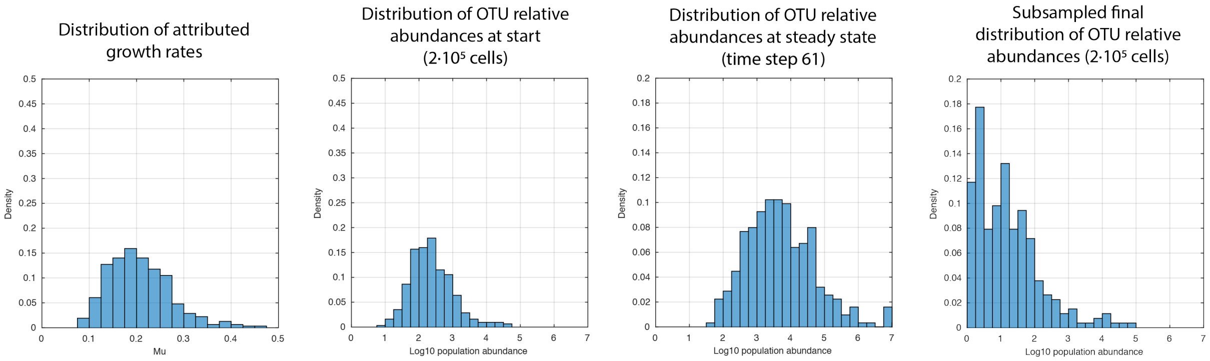

2.3 Simulation of cell death

In order to account for the possibility that cells from the sampled natural community do not grow at all because of cell death or otherwise physiological damage, we include and tested two simulations for cell death. The justification for this is that a significant amount of cells in the natural community samples stained positive for propidium iodide in flow cytometry (~75%).

In the first hypothesis, we assumed that cell death is random but equally probable for all taxa (OTUs) in the community. We removed such a cell (and the corresponding OTU) from the list that is allowed to further grow (in section 2.4).

```
%% Option 2.3.1: random cell death - this removes the OTU_id at the positions where the cell is dead.
death_rate=0.5;
death_single = rand (No_cell_community_start, 1);
death_single=double(death_single>death_rate);
OTU_id_per_Cell=death_single.*OTU_id_per_Cell;
```

In the second hypothesis, we attributed a higher probability for fast growing species to carry dead cells at time of sampling. The justification for assuming this was a result of modeling itself, namely that if abundant OTUs would be allowed to be the fastest growers, they would automatically take over the community. From the distribution of attributed growth rates, we restricted the higher dead probability to those OTUs having an initial μmax of >0.25 h^{-1} (affecting a dozen OTUs). Based on a comparison of the dead rate simulations, we concluded that the second option corresponded better to experimental observations (see figure 5b). The second option was then implemented in the pair-wise bead growth comparisons (see below Section 3.4).

```
%% Option 2.3.2: proportional cell death (85%) at time of sampling for fast growing species
(Mu>0.25). Note that this step is implemented after 2.4.1

for i=1:length(liquid_final_info.Mu);
        if liquid_final_info.Mu(i)>0.25;
        liquid_final_info.Cell_no(i)=0.15*liquid_final_info.Cell_no(i);
        end
end
```

Distribution of microcolony sizes in beads with single starter cells only

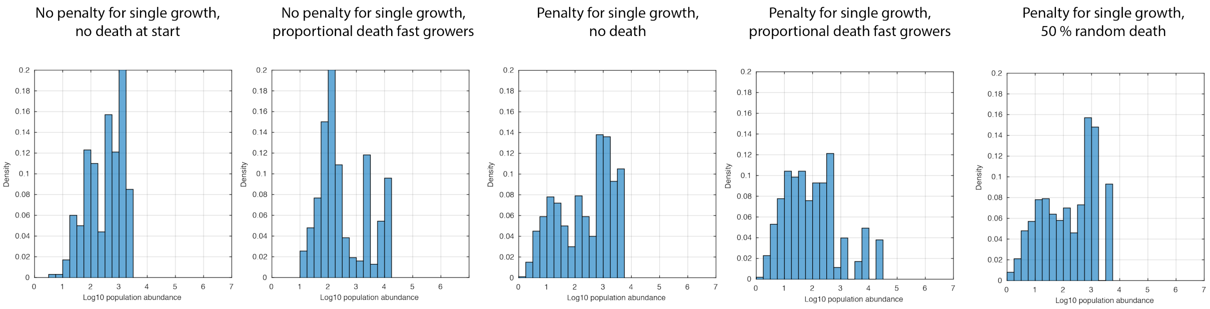

2.4 Simulation of liquid growth

The next step is to build a new table structure (liquid_final_info) with the vectors of variables and simulate those at time zero. This table is then filled with each of the randomly picked OTUs according to the probability density function.
Variables are the growth rate (Mu) at every time step, the mass of cells (M_cell), the new growth (netto_new_community), and the net number of cells per OTU (Cell_no).
The starting substrate concentration equals the one that is set by definition of the general starting parameters.

```
%2.4.1 Initialize the liquid_final_info structure

Carbon_concentration_liq(1) = initial_Carbon_concentration;

liquid_final_info.Mu(:,1) = (community_growth.Mu
*Carbon_concentration_liq(1))./(Carbon_concentration_liq(1)+Ks);
liquid_final_info.M_Cell(:,1) = biomass_per_cell*cell_distribution_per_OTU;
liquid_final_info.netto_new_community(:,1) = liquid_final_info.M_Cell(:,1)./yield;
liquid_final_info.Cell_no(:,1) = cell_distribution_per_OTU;
```

The next loop now calculates for every time step the growth for each OTU. Growth is described by Monod kinetics and includes the yield to account for CO_2 losses of carbon during growth.
It then calculates how much substrate is used (and lost in form of CO_2) in the time step, and diminishes this on the total available substrate concentration for the next time step. That substrate concentration will then influence the actual growth rate. This continues for every time step until the substrate concentration is lower than Smin. The table structure summarizes the evolution of the cell biomass, OTUs and the growth rates over all 61 time points, and the 200,000 chosen starting cells. The remaining carbon concentration is reported at every time step.

```
%2.4.2 Simulate growth

for t=2:length(Time)

    if liquid_final_info.Mu(:,t-1)>0
        liquid_final_info.M_Cell(:,t)=(liquid_final_info.M_Cell(:,t-1).*2.14.^((Time(t)-
Time(t-1))*liquid_final_info.Mu(:,t-1)));

    else
        liquid_final_info.M_Cell(:,t)=liquid_final_info.M_Cell(:,t-1);
    end
    liquid_final_info.netto_new_community(:,t) = (liquid_final_info.M_Cell(:,t) -
liquid_final_info.M_Cell(:,t-1))./yield;
```

```
    Carbon_concentration_liq(t)=Carbon_concentration_liq(t-1)-
sum(liquid_final_info.netto_new_community(:,t));
    if Carbon_concentration_liq(t)> Smin
        liquid_final_info.Mu(:,t)=(community_growth.Mu *
(Carbon_concentration_liq(t))./(Carbon_concentration_liq(t)+Ks));
    else
        liquid_final_info.Mu(:,t)=0;
    end

    liquid_final_info.Cell_no(:,t) =liquid_final_info.M_Cell(:,t)./biomass_per_cell;
end
```

The initial and final distributions of OTUs, of number of cells per OTU, their growth rates, abundances etc. can be plotted from the 'liquid_final_info' structure.

The composition of cells per OTU in steady-state (liquid_final_info.Cell_no(:,61)) is used to calculate alpha diversity measures. We subsample from this distribution to 2e5 cells to be analogous to the sequencing depth per sample. The script is rerun in at least five loops to have independent simulations and summarize diversity measures.

```
%% diversity measures
x=liquid_final_info.Cell_no(:,61);

total=sum(x);
se=round(exp(-sum(x(x>0)/total.*log(x(x>0)/total)))); %Shannon effective

sen=-sum(x(x>0)/total.*log(x(x>0)/total)); %Shannon entropy

si=sum((x(x>0)/total).^2); %Simpson concentration

sie=round(1/sum((x(x>0)/total).^2)); %Simpson effective

rich=sum(x(x>0).^0); %Richness

alpha_div=[se sen si sie rich];

T=array2table(alpha_div,'VariableNames',{'Shannon_effective','Shannon_entropy','Simpson_conce
ntration','Simpson_effective','Richness'});

writetable(T,'./Mu_liq=OTU/alpha_diversity1.csv');
```

Section 3. Low connectivity growth

In this section we describe the simulations of cell growth in beads. The basic principles are the same as before, but the assumption here is that the interactions between cells of different OTUs become more apparent when cells are confined to close proximity as in beads. We are simulating different global interaction types.
Secondly, to stay close to the experimental results, we acknowledge that beads can have a single 'occupancy' (a single starting cell per bead) or pairs of cells. This mixture grows together in the simulation, but can be differentiated on the basis of their OTU attribution in singles or pairs. We assume on average 75% of beads with single occupancy and 25% with pairs, similar to what was observed in the experiments.

The simulations start the same way as Section 1 to produce a random composition of OTUs that is weighed by a probability distribution, which is based on the measured OTU abundances in the washed soil cell suspensions. Given that $\log_{10}$ OTU-biased growth rates (section 2.2) gave better correspondence to experimental observations (Figure 3b), we continue in the remaining simulations with this way of growth rate attribution. Given further that growth-rate proportional biased cell death at start corresponded better to experimental observations (Figure 5b), we continued including this part in bead growth simulations.

First we contrast simulations in which singly occupancy has no growth penalty versus a simulation in which it has (Figure 3C). We then continue the other simulations maintaining the same growth penalty on single occupancy.

Finally, the inherent attributed OTU growth rates (as in section 2.2.1) are influenced by different types of interaction terms that are specified below. As before for high connectivity environments, we subsample the steady-state OTU or microcolony size distributions at time step 61 from five independent simulations, and compare this to experimental data (Figure 3C–F).

3.1 Attributing growth rates to cells in beads

We first attribute inherent growth rates to OTUs as in section 2.2.1 using a probability density function of $\log_{10}$ OTU relative abundances.

Next, similar to the liquid simulations we build a structure for the bead distribution of OTUs that we call beads_growth_info. This holds the information from beads with single or pairs of cells, holds their inherent growth rates and their interaction coefficients.
We start now with 200,000 beads that are filled with 1 or 2 cells. We define the percentage of beads with single cells at start (75%, based on experimental observations). This is set to '1' for simulations with beads that have single starter cells only.

```
%3.1.1 Determine proportion of beads with single and double starting members

percent_bead_single_OTU = 0.75;  %can be set to 1 when simulating single starter cells only.

% percentage of beads with a single OTU

No_beads_double_OTU = round(No_cell_community_start/(2+ (percent_bead_single_OTU/ (1-
percent_bead_single_OTU))));

% No_beads_double_OTU must be an even number!

if bitget(No_beads_double_OTU,1)
    No_beads_double_OTU=No_beads_double_OTU+1;
end

No_beads_single_OTU = No_cell_community_start - (No_beads_double_OTU *2);
total_No_bead = No_beads_single_OTU + No_beads_double_OTU;
```

The vector is randomly built, therefore we do not have to worry about the randomness of OTUs in beads. For the pairs we produce two columns, each with a randomly chosen OTU according to the initial observed OTU distribution.

```
%3.1.2. Attribute OTUs to single and double beads

beads_growth_info.single.OTU = OTU_id_per_Cell([1:No_beads_single_OTU],:);
beads_growth_info.double.OTU =
[OTU_id_per_Cell(No_beads_single_OTU:(No_beads_single_OTU+(No_beads_double_OTU/2))-
1),OTU_id_per_Cell(No_beads_single_OTU+(No_beads_double_OTU/2)+1:total_No_bead)];

No_of_bead_same_OTU=length(find([beads_growth_info.double.OTU(:,1)==
beads_growth_info.double.OTU(:,2)]));

 [s,single_bead_OTU_distribution] =
ismember(beads_growth_info.single.OTU,community_growth.OTU);
[ss,double_bead_OTU_distribution] =
ismember(beads_growth_info.double.OTU,community_growth.OTU);
```

Next we assign the maximum specific growth rates, first for beads with single OTU and then for pairs. In the first simulation scenario, there is no growth penalty on being alone in a bead. Beads with single cells have no interaction term, because they have no partner. Their starting growth rate (beads_growth_info.single.Mu) is the determining factor in the simulations.

```
%3.1.3 Keep growth rates without penalty for single cell per bead growth

beads_growth_info.single.Mu = community_growth.Mu(single_bead_OTU_distribution);

%this gives us 1 column vector

beads_growth_info.double.Mu = community_growth.Mu(double_bead_OTU_distribution);

%this gives us two columns, column 1 for the first partner, column 2 for the other.
```

In the alternative scenario, we impose a penalty on the attributed inherent growth rates when cells are alone as starters in individual beads. The penalty is higher when the inherent growth rate is slower. Simulation results indicated that this second scenario of single-starter growth penalty is better explaining experimental observations of single starter bead growth (e.g., figure 5b). Therefore, the growth penalty is kept in all subsequent paired-growth bead simulations.

```
% 3.1.4. Penalty for growth as a single starter cell in a bead, which is inverse proportional
to the attributed inherent OTU growth rate.

single_community_growth.Mu = community_growth.Mu .*
(1.2./(community_growth.Mu.^log10(community_growth.Mu)));

beads_growth_info.single.Mu = single_community_growth.Mu(single_bead_OTU_distribution);

beads_growth_info.double.Mu = community_growth.Mu(double_bead_OTU_distribution);
```

Cell death is again implemented as before, being more severe for OTUs with assigned growth rates above 0.25 h^{-1}.

```
%% 3.1.5 Proportional cell death for fast growing species

death_single = ones(No_beads_single_OTU, 1);

for i=1:length(beads_growth_info.single.Mu);
        if beads_growth_info.single.Mu(i)>0.25;
                if rand>0.15;
                death_single(i) = 0;
                else
                death_single(i) = 1;
                end
        end
end

Bead_final_info.single.Cell_no = death_single.* Bead_final_info.single.Cell_no;
Bead_final_info.single.M_Cell =
Bead_final_info.single.M_Cell.*Bead_final_info.single.Cell_no;
```

3.2 Attributing interaction effects to OTU pairs in beads

With paired OTUs inside individual beads we allow OTU growth rates to be influenced by an interaction parameter, for which we test different scenarios, having either totally random influence, biased positive or negative influence, or bimodal influence, as specified below. We contrast these simulations to a ‘null’ model, in which there are no interactions at all.

To accommodate interaction strengths, we create a parameter ‘community_growth.interaction’ that influences (by simple multiplication) the assigned growth rates. The growth interaction parameter is assigned at the simulation start and is different for the different interaction scenarios, but remains the same at all simulation time steps.

3.3 Initializing the bead growth simulation in the null model (no interactions).

Next we create a new structure for the actual growth simulation named 'Bead_final_info' that will collect the growth of cells and OTUs in the beads with single or double starting occupancy. This is similar as section 2.3 for liquid growth.

```
%3.3.1 Initialize the Bead_final_info growth simulation structure

%% Building vectors of variables and simulating time zero values

% Compute all the variables for t = 0. The later time points will be calculated in the loop
Carbon_concentration_bead(1) = initial_Carbon_concentration;

% Growth described by Monod kinetics
% Building up bead properties at time=zero
% beads_growth_info.interaction parameter affects the Mu and consequently
% number of geneRATED cells

Bead_final_info.single.Mu(:,1)= (beads_growth_info.single.Mu *
Carbon_concentration_bead(1))./(Carbon_concentration_bead(1)+ Ks);

Bead_final_info.single.M_Cell(1:length(beads_growth_info.single.OTU),1) = biomass_per_cell;
Bead_final_info.single.netto_new_bead(1:length(beads_growth_info.single.OTU),1)=
Bead_final_info.single.M_Cell(:,1)./yield;
Bead_final_info.single.Cell_no(1:length(beads_growth_info.single.OTU),1) = 1;

Bead_final_info.double.Mu_1(:,1) = beads_growth_info.double.interaction(:,1)
.*(beads_growth_info.double.Mu(:,1) *
Carbon_concentration_bead(1))./(Carbon_concentration_bead(1)+ Ks);
Bead_final_info.double.Mu_2(:,1) = beads_growth_info.double.interaction(:,2)
.*(beads_growth_info.double.Mu(:,2) *
Carbon_concentration_bead(1))./(Carbon_concentration_bead(1)+ Ks);

for k=1:length(Bead_final_info.double.Mu_1)
    if Bead_final_info.double.Mu_1(k,1)> Mu_max
        Bead_final_info.double.Mu_1(k,1)= (0.25 .* rand(1)+0.75).*Mu_max;
    end
end
for k=1:length(Bead_final_info.double.Mu_2)
    if Bead_final_info.double.Mu_2(k,1)> Mu_max
        Bead_final_info.double.Mu_2(k,1)= (0.25.*rand(1)+0.75).*Mu_max;
    end
end

Bead_final_info.double.M_Cell_1(1:length(beads_growth_info.double.OTU),1) = biomass_per_cell;
Bead_final_info.double.M_Cell_2(1:length(beads_growth_info.double.OTU),1) = biomass_per_cell;

Bead_final_info.double.netto_new_bead(1:length(beads_growth_info.double.OTU),1)=
(Bead_final_info.double.M_Cell_1(:,1)+Bead_final_info.double.M_Cell_2(:,1))./yield;

Bead_final_info.double.Cell_no_1(1:length(beads_growth_info.double.OTU),1) = 1;
Bead_final_info.double.Cell_no_2(1:length(beads_growth_info.double.OTU),1) = 1;
```

At this point, we again include proportional cell death for fast-growing species.

```
%3.3.2 Introduce proportional death rate for Mu > 0.25, there is 85% chance to be a dead cell

death_single = ones(No_beads_single_OTU, 1);

for i=1:length(beads_growth_info.single.Mu);
       if beads_growth_info.single.Mu(i)>0.25;
              if rand>0.15;
              death_single(i) = 0;
              else
              death_single(i) = 1;
              end
       end
end

Bead_final_info.single.Cell_no = death_single.* Bead_final_info.single.Cell_no;
Bead_final_info.single.M_Cell =
Bead_final_info.single.M_Cell.*Bead_final_info.single.Cell_no;

%same thing for the OTUs in pairs

death_double = ones(No_beads_double_OTU/2, 2);
```

```
for i=1:length(beads_growth_info.double.Mu);
        if beads_growth_info.double.Mu(i,1)>0.25;
                if rand>0.15;
                death_double(i,1) = 0;
                else
                death_double(i,1) = 1;
                end
        end
        if beads_growth_info.double.Mu(i,2)>0.25;
                if rand>0.15;
                death_double(i,2) = 0;
                else
                death_double(i,2) = 1;
                end
        end

end


Bead_final_info.double.Cell_no_1 = death_double(:,1).*Bead_final_info.double.Cell_no_1;
Bead_final_info.double.Cell_no_2 = death_double(:,2).*Bead_final_info.double.Cell_no_2;

%multiply to determine the start viable biomass

Bead_final_info.double.M_Cell_1=
Bead_final_info.double.M_Cell_1.*Bead_final_info.double.Cell_no_1;

Bead_final_info.double.M_Cell_2=
Bead_final_info.double.M_Cell_2.*Bead_final_info.double.Cell_no_2;
```

The next loop now calculates for every time step the growth for each OTU. As in Section 2.3, growth is described by Monod kinetics and includes the yield to account for CO_2 losses of carbon during growth.

The simulation then calculates how much substrate is used (on the basis of the yield, and lost in form of CO_2), and diminishes this on the total available substrate concentration for the next round. That substrate concentration will then influence the actual growth rate. This continues for every time step until the substrate concentration is lower than S_{min}. The table structure summarizes the evolution of the cell biomass, OTUs, and growth rates over all 61 time points.

```
%3.3.3 Simulate growth in beads for single and double occupancy

for t=2:length(Time)
    if Bead_final_info.single.Mu(:,t-1)>0
        Bead_final_info.single.M_Cell(:,t)=Bead_final_info.single.M_Cell(:,t-
1).*2.14.^((Time(t)-Time(t-1))*Bead_final_info.single.Mu(:,t-1));
    else
        Bead_final_info.single.M_Cell(:,t)=Bead_final_info.single.M_Cell(:,t-1);
    end


    if Bead_final_info.double.Mu_1(:,t-1)>0
        Bead_final_info.double.M_Cell_1(:,t)=Bead_final_info.double.M_Cell_1(:,t-
1).*2.14.^((Time(t)-Time(t-1))*Bead_final_info.double.Mu_1(:,t-1));
    else
        Bead_final_info.double.M_Cell_1(:,t)=Bead_final_info.double.M_Cell_1(:,t-1);
    end

    if Bead_final_info.double.Mu_2(:,t-1)>0
        Bead_final_info.double.M_Cell_2(:,t)=Bead_final_info.double.M_Cell_2(:,t-
1).*2.14.^((Time(t)-Time(t-1))*Bead_final_info.double.Mu_2(:,t-1));
    else
        Bead_final_info.double.M_Cell_2(:,t)=Bead_final_info.double.M_Cell_2(:,t-1);
    end

    Bead_final_info.single.netto_new_bead(:,t)= (Bead_final_info.single.M_Cell(:,t)-
Bead_final_info.single.M_Cell(:,t-1))./yield;
    Bead_final_info.double.netto_new_bead(:,t)= ((Bead_final_info.double.M_Cell_1(:,t) +
Bead_final_info.double.M_Cell_2(:,t)) - (Bead_final_info.double.M_Cell_1(:,t-
1)+Bead_final_info.double.M_Cell_2(:,t-1)))./yield;

    % the remaining carbon is what was before minus what was consumed/spent for
    % new growth of community
```

```
    Carbon_concentration_bead(t)=Carbon_concentration_bead(t-1)-
sum(Bead_final_info.single.netto_new_bead(:,t))-
sum(Bead_final_info.double.netto_new_bead(:,t));

    if Carbon_concentration_bead(t)>Smin
        Bead_final_info.single.Mu(:,t)= (beads_growth_info.single.Mu *
(Carbon_concentration_bead(t))).((Carbon_concentration_bead(t)+Ks));
        Bead_final_info.double.Mu_1(:,t)= Bead_final_info.double.Mu_1(:,1) *
(Carbon_concentration_bead(t))./((Carbon_concentration_bead(t)+ Ks));
        Bead_final_info.double.Mu_2(:,t)= Bead_final_info.double.Mu_2(:,1) *
(Carbon_concentration_bead(t))./((Carbon_concentration_bead(t)+ Ks));
    else
        Bead_final_info.single.Mu(:,t)=0;
        Bead_final_info.double.Mu_1(:,t)=0;
        Bead_final_info.double.Mu_2(:,t)=0;
    end
    Bead_final_info.single.Cell_no(:,t) =
Bead_final_info.single.M_Cell(:,t)/biomass_per_cell;
    Bead_final_info.double.Cell_no_1(:,t) =
Bead_final_info.double.M_Cell_1(:,t)/biomass_per_cell;
    Bead_final_info.double.Cell_no_2(:,t) =
Bead_final_info.double.M_Cell_2(:,t)/biomass_per_cell;
end
```

Finally, we sum the OTUs per bead at the end of the simulations to compare their abundance distribution to that of liquid, or of the experimental observations (Figure 3).

```
%% 3.3.3 Sum for each OTU the cells across all beads, first for partner 1, then for partner
2, and separately for beads starting with a single cell.

for i=1:No_OTUs
       A=double_bead_OTU_distribution(:,1)==i;
       B=Bead_final_info.double.Cell_no_1(:,61);
       C=B(A);
       Bead_final_info.double.OTU_cells(1,i)=sum(C);
    Bead_final_info.double.OTU_cells(2,i)=mean(C);
    Bead_final_info.double.OTU_cells(3,i)=std(C);

       D=double_bead_OTU_distribution(:,2)==i;
       E=Bead_final_info.double.Cell_no_2(:,61);
       F=E(D);
       Bead_final_info.double.OTU_cells(4,i)=sum(F);
    Bead_final_info.double.OTU_cells(5,i)=mean(F);
    Bead_final_info.double.OTU_cells(6,i)=std(F);

    G=single_bead_OTU_distribution(:,1)==i;
    H=Bead_final_info.single.Cell_no(:,61);
    HH=H(G);
    Bead_final_info.single.OTU_cells(1,i)=sum(HH);
    Bead_final_info.single.OTU_cells(2,i)=mean(HH);
    Bead_final_info.single.OTU_cells(3,i)=std(HH);
end

Bead_combined_productivity=Bead_final_info.single.OTU_cells(1,:)+Bead_final_info.double.OTU_c
ells(1,:)+Bead_final_info.double.OTU_cells(4,:);

Bead_final_info_double =
Bead_final_info.double.OTU_cells(1,:)+Bead_final_info.double.OTU_cells(4,:);
```

For the OTU diversity analysis, we subsample from the results at time step 61 (i.e., 'Bead_final_info.double.OTU_cells').

```
%%3.3.4 subsampling for the final plots, as if it is sequencing with max 2e5 reads per sample
% sample the final cell composition at time 61

B=Bead_combined_productivity;
probability_vector_combined=100*(B/sum(B)); %make new probability vector based on final
distribution
OTU_end=datasample(community_growth.OTU,No_cell_community_start,'Weights',probability_vector_
combined); %subsample the OTUs according to the final probability
[cell_distribution_per_OTU_combined_end,b] = histc(OTU_end,OTU_Value(:,1));

Double=Bead_final_info_double;
```

```
probability_vector_double=100*(Double/sum(Double)); %make new probability vector based on
final distribution
OTU_end_double=datasample(community_growth.OTU,No_cell_community_start,'Weights',probability_
vector_double); %subsample the OTUs according to the final probability
[cell_distribution_per_OTU_double_end,bb] = histc(OTU_end_double,OTU_Value(:,1));

Single=Bead_final_info.single.OTU_cells(1,:);
probability_vector_single=100*(Single/sum(Single)); %make new probability vector based on
final distribution
OTU_end_single=datasample(community_growth.OTU,No_cell_community_start,'Weights',probability_
vector_single); %subsample the OTUs according to the final probability
[cell_distribution_per_OTU_single_end,bbb] = histc(OTU_end_single,OTU_Value(:,1));
```

Distribution of OTUs in beads without interactions or starter cell death (null model)

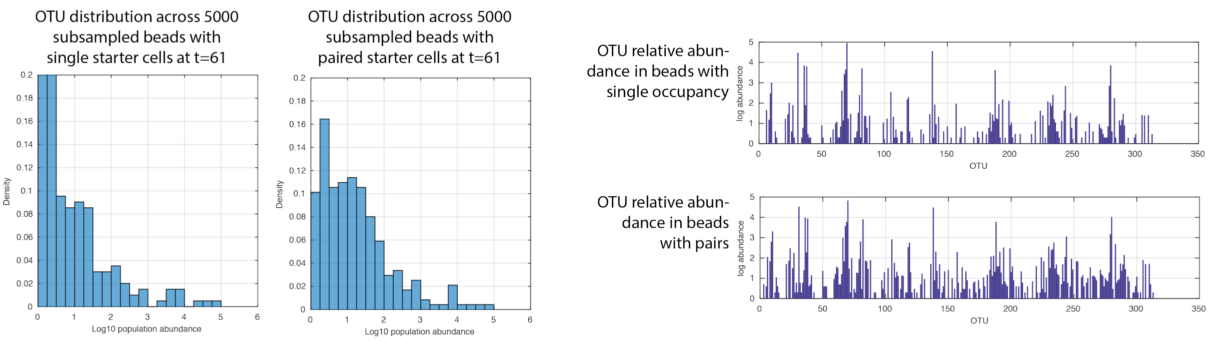

To simulate quantified bead growth, we remove cells that have not grown, subsample to the amount of observed beads and quantify paired interactions along a 2D grid.

```
%% 3.3.5 Remove pairs which are both dead and quantify growth interactions from scatter
diagram

%include beads with 'zero' in the plots for those that have death rates
%replace '0' by '1' in the last column

partner_1=Bead_final_info.double.Cell_no_1(:,61);
partner_2=Bead_final_info.double.Cell_no_2(:,61);

partner_1(partner_1==0)=1;
partner_2(partner_2==0)=1;

double_bead_productivity=[partner_1 partner_2];

%remove pairs which both are dead (==1 in both columns)

sum_DB=sum(double_bead_productivity,2);
dead=sum_DB==2;
dead=repelem(dead,1,2);
ZZ=double_bead_productivity(~dead);
double_bead_productivity_clean=reshape(ZZ,[],2);


double_bead_prod_subsample=datasample(double_bead_productivity_clean,5000);

%% count interactions from scatter diagram paired growth ratios

%1.count where one partner is dead. This would essentially be the situation of a 'single
occupancy'.

scat=double_bead_prod_subsample;
B=scat(:,1)==1 & scat(:,2)>1 | scat(:,1)>1 & scat(:,2)==1;
B=repelem(B,1,2);
Bpeak=scat(B);
s=reshape(Bpeak,[],2);
dead=length(s)

fraction_single=dead/length(scat)

%2.count where one partner is inhibited. First determine whether one of the partners has a
value ==1. These we would have to removed. Recover the logic double from the previous
element, but now in inverse. Then calculate the ratio.
```

```
scat_double=scat(~B);
scat_double=reshape(scat_double,[],2);
scat_div=scat_double(:,1)./scat_double(:,2);

%if < 1, then invert

for i=1:length(scat_div);

if scat_div(i)<1
        scat_div(i)=1/scat_div(i);
end
end

%grid count

LG=log10(scat_double);
anchor=[0,0;6,6];
LG=vertcat(anchor, LG);
nbins=[12 12];
p1=hist3((LG),nbins);
p1=reshape(p1,[],1);

p1=array2table(p1,'VariableNames',{'grid_count'});
writetable(p1,'grid_count1.csv');

%plot this histogram and extract data

FigH=figure;
height=100;
width=100;
x0=10;
y0=10;
p_1=histogram(log10(scat_div),'Normalization','probability');
p_1.BinWidth=0.25;
counts_pv_1=p_1.Values;
bin_pv_1=p_1.NumBins;
lim_pv_1=p_1.BinLimits;
set(gca,'fontsize',6)
xlim([0,7]);
ylim([0,0.5]);
set(gcf,'position',[x0,y0,width,height]);
xlabel('log10 ratio', 'FontSize',6)
ylabel('density', 'FontSize',6)
grid on
filename=sprintf('double-subsample-interaction-ratio-histogram.pdf');
title(filename);
saveas(FigH, filename,'pdf');


hist_1=vertcat(dead, fraction_single,bin_pv_1,lim_pv_1',counts_pv_1');

P=array2table(hist_1,'VariableNames',{'bins_interactions'});
writetable(P,'interaction_bins1.csv');
```

Interaction vectors are summarized from five independent simulations and compared between different simulations.

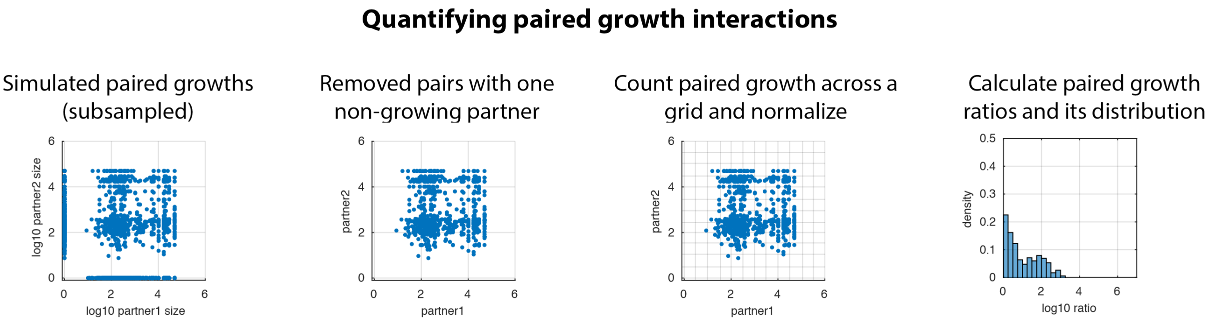

3.4 Imposing different interaction effects to both partner in every bead

To conceptually simulate the effect of interspecific interaction types we maintained the general growth penalty to single occupancy and the proportional cell dead to fast-growing species in all further simulations.

3.4.1 Bimodal interaction coefficients

In the first simulation of interspecific interactions we assumed that there may be a subset of taxa that gain an overproportional profit at the expense of the others. We call this simulation a **bimodal** interaction effect. We create the bimodality as two distributions, from which we subsequently sample the interaction coefficient.
First we attribute an inherent interaction parameter to the OTUs. Then, we impose a different bimodal random influence on the **inherent** interaction parameters in pairs, with on average in 40% of cases a negative and 60% of cases a positive influence. The final interaction parameter in pairs will influence the attributed growth rates of each OTU in a pair. The model is then continued as for sections 3.3 above to simulate growth and calculate steady-state community diversity.

```
%% 3.4.1 Bimodal interactions

%% make two probability distribution curves and chose inherent interaction strength.

pd1=makedist('Lognormal','mu',0.1,'sigma',1.0);
pd2=makedist('Normal','mu',0.7,'sigma',0.1);

for i=1:No_OTUs
    if community_growth.OTU_Value(i) < 630 %an arbitrary value to penalize a majority of
attributed slower-growing OTUs.
        community_growth.interaction(i,1)=random(pd1)/6;
    else
        community_growth.interaction(i,1)=random(pd2);
    end
end

% building beads_growth_info first for those with single OTU and then for pairs

single_community_growth.Mu = community_growth.Mu .*
(1.2./(community_growth.Mu.^log10(community_growth.Mu))); %penalty on single growth. The more
slower the inherent growth rate the higher the penalty.

beads_growth_info.single.Mu = single_community_growth.Mu(single_bead_OTU_distribution); %this
gives us 1 column vector


beads_growth_info.double.Mu = community_growth.Mu(double_bead_OTU_distribution); %this gives
us two columns, column 1 for the first partner, column 2 for the other.
beads_growth_info.double.interaction =
community_growth.interaction(double_bead_OTU_distribution); %this gives us two columns,
column 1 for the first partner, column 2 for the other.

%% create the bimodal double bead interaction term

percentage_interaction_model_1= 0.60;
model_1=(random(pd1,[1,round(percentage_interaction_model_1*No_beads_double_OTU)]))/20;
[A_1,B_1]=histcounts(model_1,round(percentage_interaction_model_1*No_beads_double_OTU));

model_2=(random(pd2,[1,(No_beads_double_OTU-length(model_1))]));
[A_2,B_2]=histcounts(model_2,No_beads_double_OTU-length(model_1));
bimodal=[B_1 B_2]';

OTU_bimodal_effect_1=datasample(bimodal,(No_beads_double_OTU/2));
OTU_bimodal_effect_2=datasample(bimodal,(No_beads_double_OTU/2));


beads_growth_info.double.interaction(:,1)=beads_growth_info.double.interaction(:,1).*OTU_bimo
dal_effect_1;
beads_growth_info.double.interaction(:,2)=beads_growth_info.double.interaction(:,2).*OTU_bimo
dal_effect_2;

%from here, continue as in section 3.3.1
```

Subsampled microcolony size distribution across beads with single starter cell at t=61

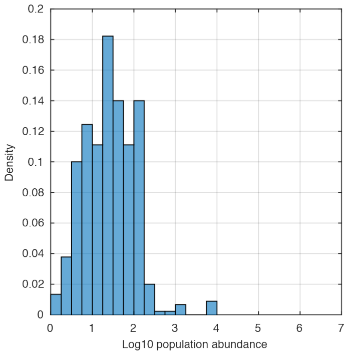

Subsampled microcolony size distribution across beads with paired growth at t=61

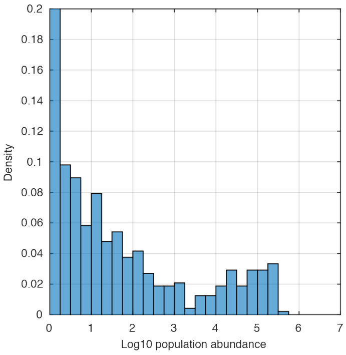

Subsampled OTU relative abundance in beads with single starter cell at t=61

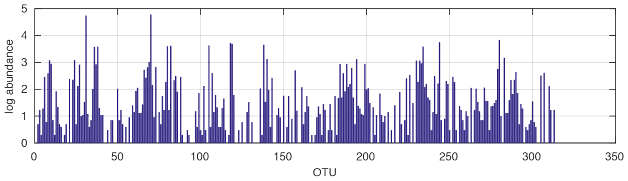

Subsampled OTU relative abundance in beads with paired starter cells at t=61

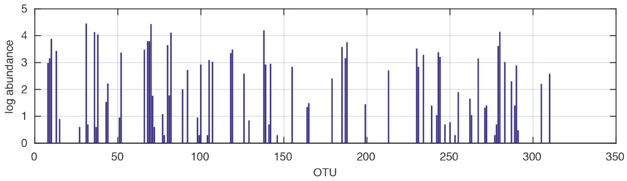

Subsampled OTU relative abundance âcross all beads at t=61 (75% beads with single, 25% with starting pairs)

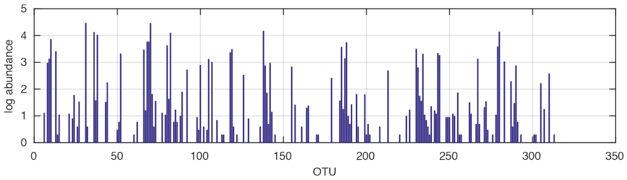

3.4.2 Biased random positive and negative interactions

In the second interspecific interaction scenario, we impose on each of the partners in a pair **independently** a 40% chance for a random negative penalty to the growth rate, and a 60% chance for a random positive effect. Section 3.4.1 is removed.

```
%% 3.4.2. Loop to randomly create a 40% change for a negative and 60% chance for a positive
interaction on growth in double bead occupancy.


for j=1:length(beads_growth_info.double.Mu_1);
    if rand >0.6;
    beads_growth_info.double.Mu_1(j) = (0.2.*rand(1) + 0.4).* beads_growth_info.double.Mu_1
(j);
    else
    beads_growth_info.double.Mu_1(j) = (0.8.*rand(1) + 0.6).* beads_growth_info.double.Mu_1
(j);
    end
end
for j=1:length(beads_growth_info.double.Mu_2);
    if rand >0.6;
    beads_growth_info.double.Mu_2(j) = (0.2.*rand(1) + 0.4).* beads_growth_info.double.Mu_2
(j);
    else
    beads_growth_info.double.Mu_2(j) = (0.8.*rand(1) + 0.6).* beads_growth_info.double.Mu_2
(j);
    end
end
beads_growth_info.double.Mu=[beads_growth_info.double.Mu_1 beads_growth_info.double.Mu_2];

%from here continue as in section 3.3.1
```

Subsampled microcolony size distribution across beads with single starter cell at t=61

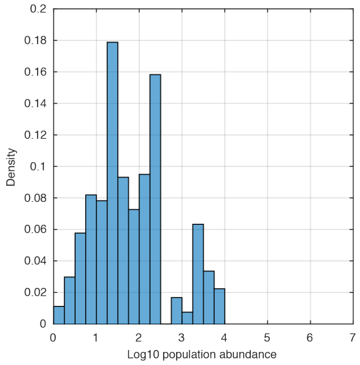

Subsampled microcolony size distribution across beads with paired growth at t=61

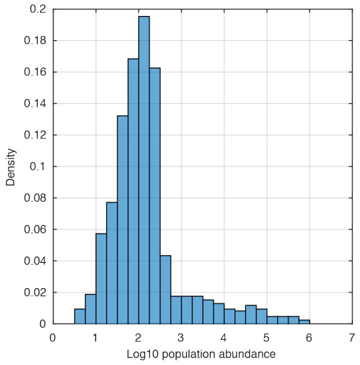

Subsampled OTU relative abundance in beads with single starter cell at t=61

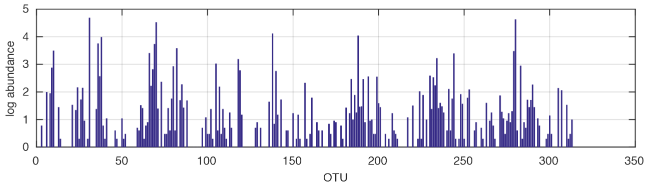

Subsampled OTU relative abundance in beads with paired starter cells at t=61

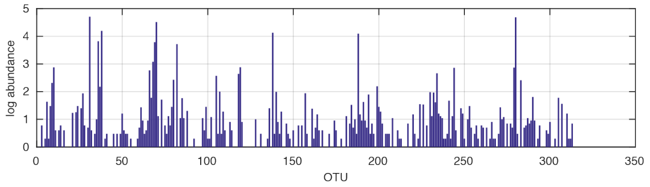

Subsampled OTU relative abundance âcross all beads at t=61 (75% beads with single, 25% with starting pairs)

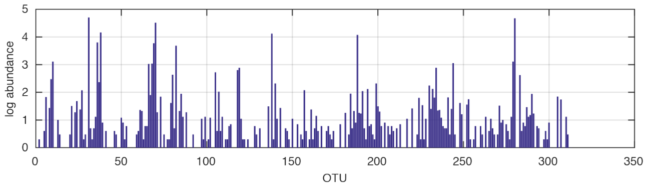

3.4.3 Random positive influence on slow growers

In the third interspecific interaction model, we assume that slow growers in 40% of the cases are on average favored more by having a partner than are fast growers. The influence takes the form of a multiplication of the attributed growth rate by the –log (in Matlab terms, the natural logarithm) of that growth rate. We include a further condition that the maximum µmax cannot be surpassed.

```
%% 3.4.3. Random positive influence curve for slow growers
%% by multiplying with the -log of the respective Mu.

beads_growth_info.double.Mu_1=beads_growth_info.double.Mu(:,1);
beads_growth_info.double.Mu_2=beads_growth_info.double.Mu(:,2);

%creating the loop for attribution of the positive influence

for j=1:length(beads_growth_info.double.Mu_1);
    if rand >0.6;
    beads_growth_info.double.Mu_1(j) = -log(beads_growth_info.double.Mu_1(j)).*
beads_growth_info.double.Mu_1 (j);
    if beads_growth_info.double.Mu_1(j) > Mu_max;
    beads_growth_info.double.Mu_1(j) = Mu_max;
    end
    else
    beads_growth_info.double.Mu_1(j) = beads_growth_info.double.Mu_1 (j);
    end
end
for j=1:length(beads_growth_info.double.Mu_2);
    if rand >0.6;
    beads_growth_info.double.Mu_2(j) = -log(beads_growth_info.double.Mu_2(j)).*
beads_growth_info.double.Mu_2 (j);
    if beads_growth_info.double.Mu_2(j) > Mu_max;
    beads_growth_info.double.Mu_2(j) = Mu_max;
    end
    else
```

```
    beads_growth_info.double.Mu_2(j) = beads_growth_info.double.Mu_2 (j);
    end
end
beads_growth_info.double.Mu=[beads_growth_info.double.Mu_1 beads_growth_info.double.Mu_2];

%from here we continue with section 3.3.1.
```

Subsampled microcolony size distribution across beads with single starter cell at t=61

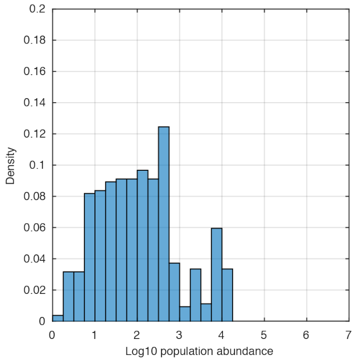

Subsampled microcolony size distribution across beads with paired growth at t=61

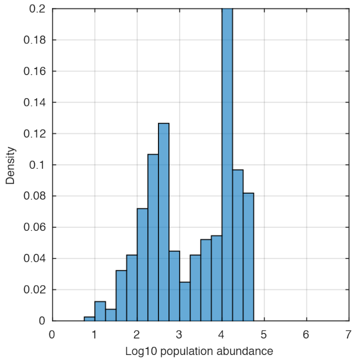

Subsampled OTU relative abundance in beads with single starter cell at t=61

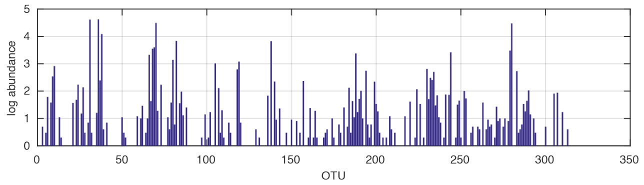

Subsampled OTU relative abundance in beads with paired starter cells at t=61

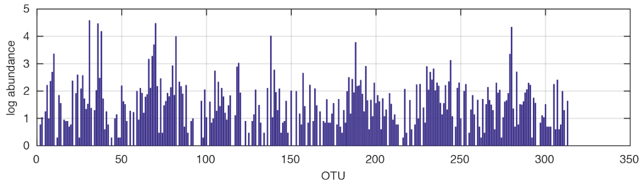

Subsampled OTU relative abundance âcross all beads at t=61 (75% beads with single, 25% with starting pairs)

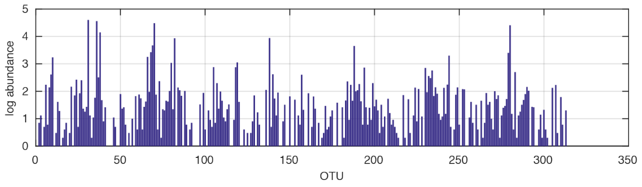

3.4.4 Random interaction

In the fourth interspecific interaction scenario, we simulate a interaction factor that affects each cell of each OTU in a pair independently. The factor is drawn randomly within the range of 0.01–1.25 and is used to multiply with the attributed starting growth rates. It can thus mostly reduce but also improve growth rates.

```
%% 3.4.4 Creating random influence curve for the double bead interaction term in the interval
(0.01-1.25)
% growth rate will be affected randomly for every OTU in every pair,
% independent of the OTU

beads_growth_info.double.interaction=(0.01+1.25.*rand(No_beads_double_OTU/2,2));

%from here we continue with section 3.3.1
```

Subsampled microcolony size distribution across beads with single starter cell at t=61

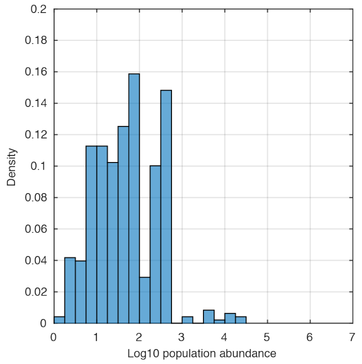

Subsampled microcolony size distribution across beads with paired growth at t=61

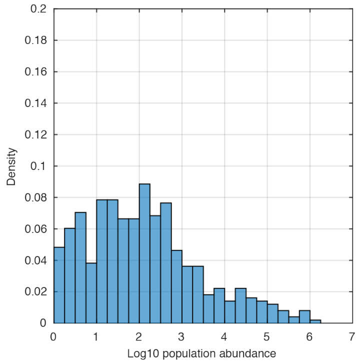

Subsampled OTU relative abundance in beads with single starter cell at t=61

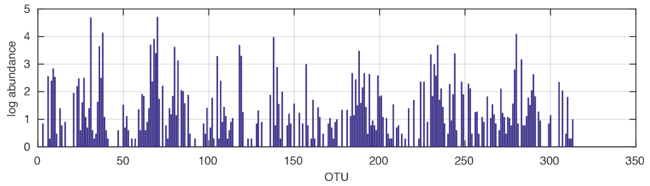

Subsampled OTU relative abundance in beads with paired starter cells at t=61

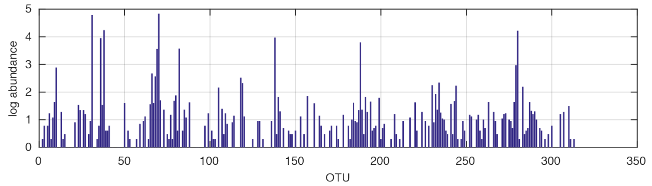

Subsampled OTU relative abundance âcross all beads at t=61 (75% beads with single, 25% with starting pairs)

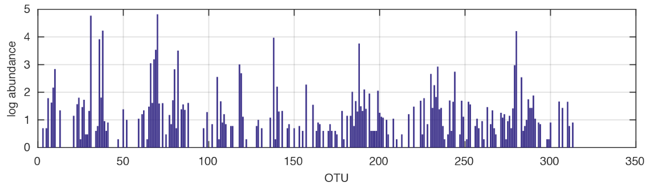

3.4.5 Biased growth penalty on slow growers

Finally, in the last scenario we assume that slow growers on average are negatively influenced in a paired relationship. We attribute this interaction in form of a penalty on growth rates in pairs, imposed for each partner OTU independently. Faster growers have a smaller chance to be influenced by this penalty than OTUs with slower growth rates.

```
%% 3.4.4 Single growth penalty; biased interaction penalty to slow growers
%% creating the chance influence on growth; faster growers have smaller chance to be
influenced by interactions

for j=1:length(beads_growth_info.double.Mu_1);
    if beads_growth_info.double.Mu_1 > 0.15;
        if rand >0.8;
            beads_growth_info.double.Mu_1(j) = (0.01*rand + 0.09).*
beads_growth_info.double.Mu_1(j);
        else
            beads_growth_info.double.Mu_1(j) = beads_growth_info.double.Mu_1(j);
        end
    else
        if rand >0.6;
                beads_growth_info.double.Mu_1(j) = (0.01*rand + 0.09).*
beads_growth_info.double.Mu_1(j);
        else
            beads_growth_info.double.Mu_1(j) = beads_growth_info.double.Mu_1(j);
        end
    end
end

for j=1:length(beads_growth_info.double.Mu_2);
    if beads_growth_info.double.Mu_2 > 0.15;
        if rand >0.8;
            beads_growth_info.double.Mu_2(j) = (0.01*rand + 0.09).*
beads_growth_info.double.Mu_2(j);
        end
```

```
    else
        if rand >0.6;
            beads_growth_info.double.Mu_2(j) = (0.01*rand + 0.09).*
beads_growth_info.double.Mu_2(j);
        end
    end
end

beads_growth_info.double.Mu=[beads_growth_info.double.Mu_1 beads_growth_info.double.Mu_2];

%from here we continue with section 3.3.1
```

Subsampled microcolony size distribution across beads with single starter cell at t=61

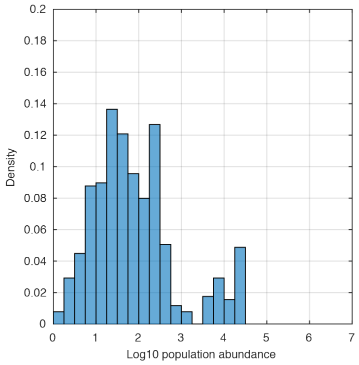

Subsampled microcolony size distribution across beads with paired growth at t=61

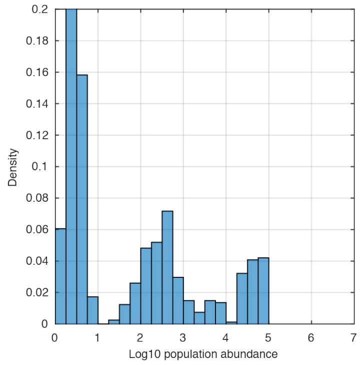

Subsampled OTU relative abundance in beads with single starter cell at t=61

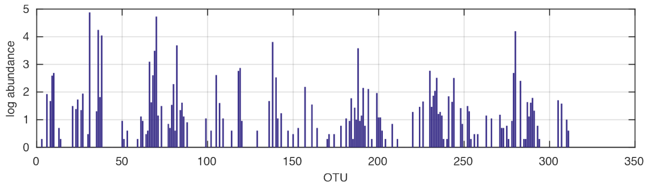

Subsampled OTU relative abundance in beads with paired starter cells at t=61

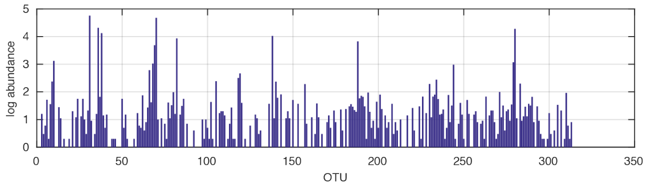

Subsampled OTU relative abundance âcross all beads at t=61 (75% beads with single, 25% with starting pairs)

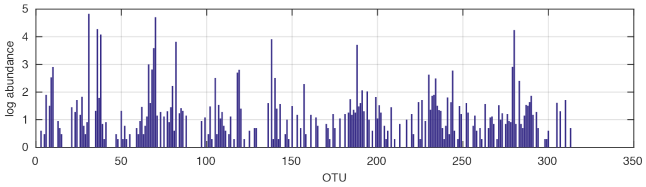

Section 4. Preparation of agarose beads with encapsulated cells.

Materials:

1. Cell suspension with around 10^7 to 10^8 cells per ml (diluted in MOPS or PBS)
2. Agarose 1% (low melting)
3. Pluronic acid: Pluronic F-68 solution 10% Gibco
4. Silicone oil (Dimethylpolysiloxane by Sigma, DMPS5X-500G)
5. 1x PBS
6. MOPS or PBS buffer without C-Source
7. 10, 25 ml glass test tubes
8. 50 ml falcon tubes
9. Set pipette: 30µl, 200µl, 1ml
10. Vortex
11. Centrifuge (Swing bucket rotors)
12. Timer
13. Ice bath
14. 40, 70–µm filter strainers (Corning).

Prepare:

- Fill the 25ml glass tubes with 15ml of silicon oil. Prewarm to 37ºC.
- Prewarm pluronic acid, tubes, tips to 42ºC
- Dissolve Agarose. Let it cool down to a suitable temperature for the cells (max 37°C)

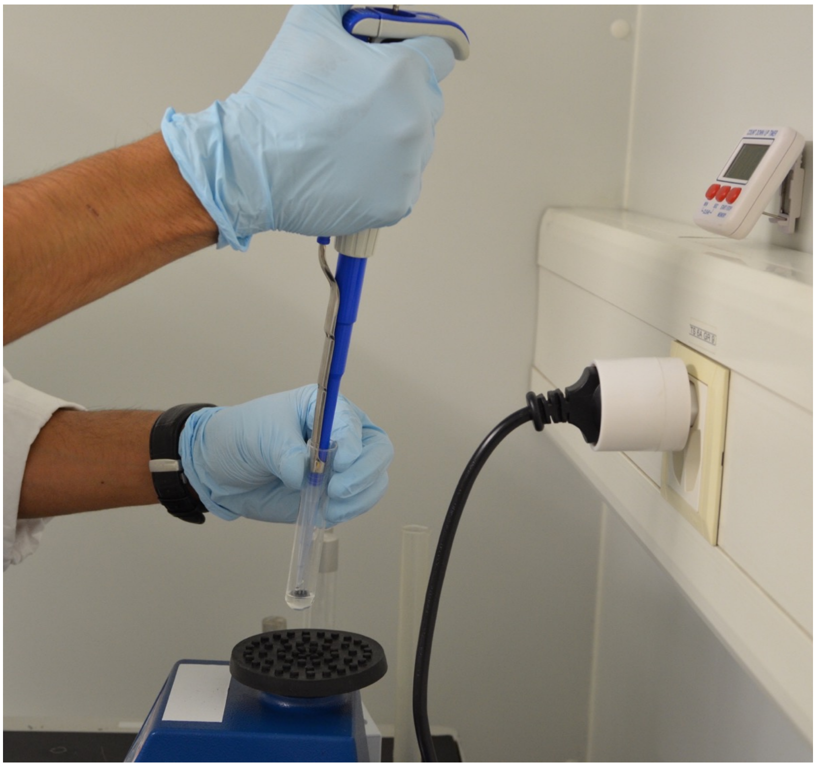

- (This part of the work can be carried out in a 37°C room)
- Add 1 ml of agarose to 30 µl pluronic acid in 10 ml glass tube
- Make sure that the temperature of agarose is optimal for the cells

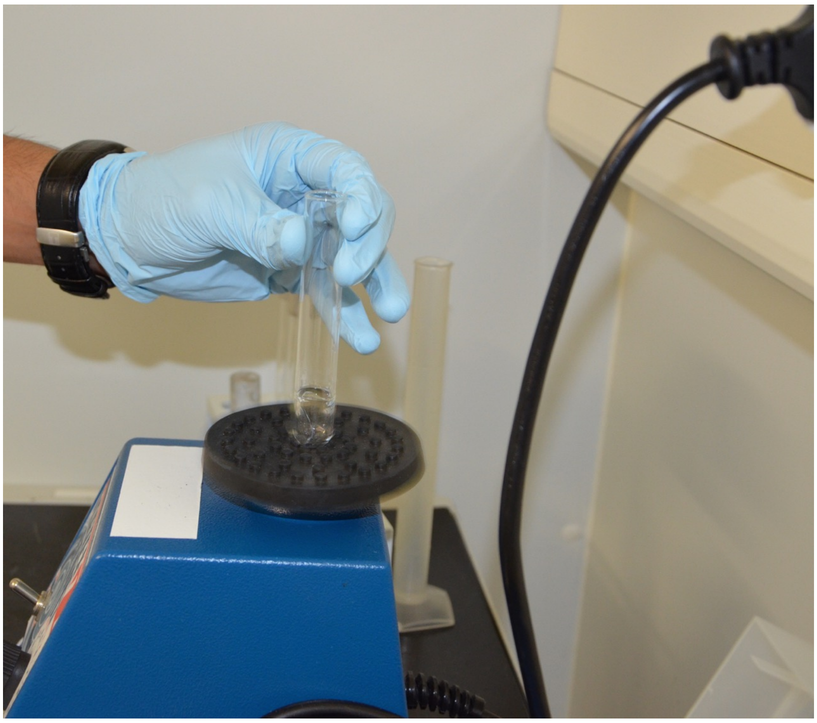

- vortex vigorously for 1 minute

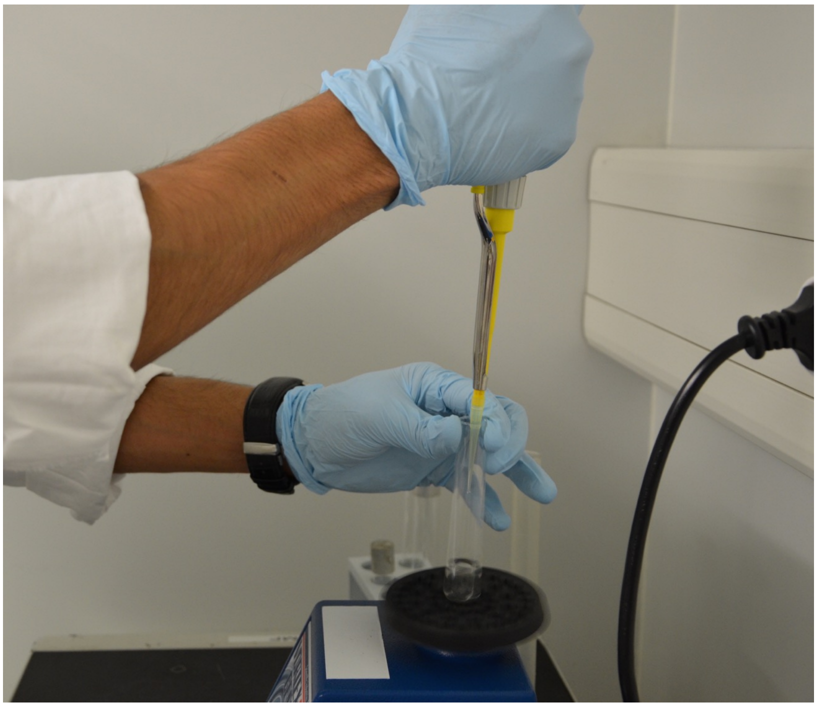

- Add 200 µl of cells and vortex again for 1 minute

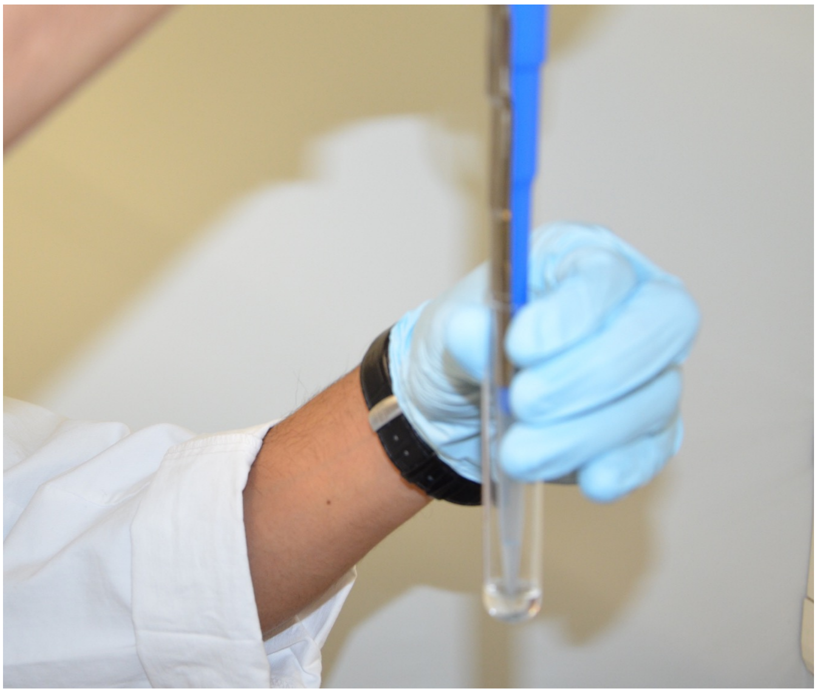

- Pipette 500 µl of the mixture

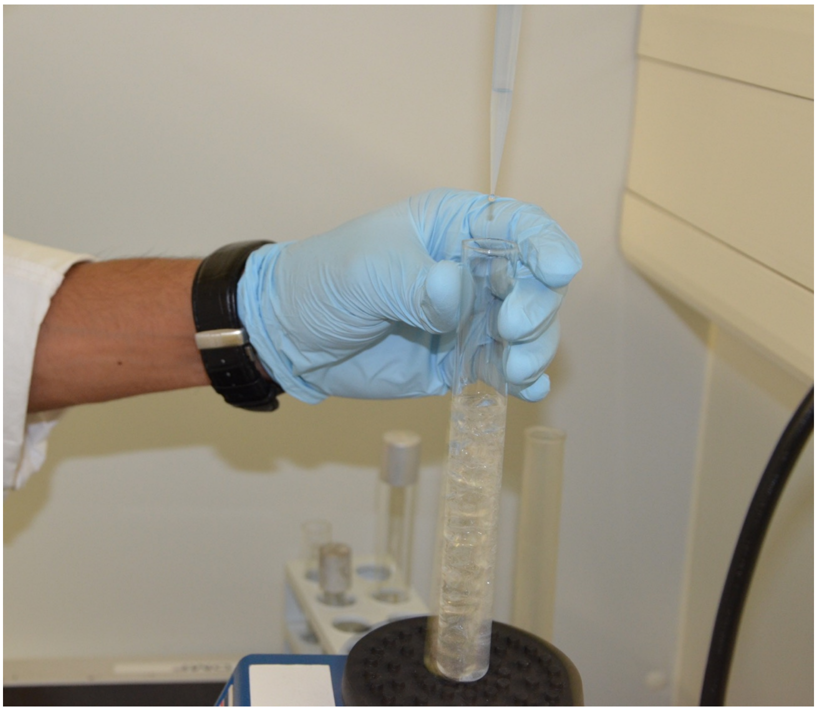

- Add the mixture slowly to the oil while vortexing
- Vortex vigorously for 2 minutes to form the beads (max speed)

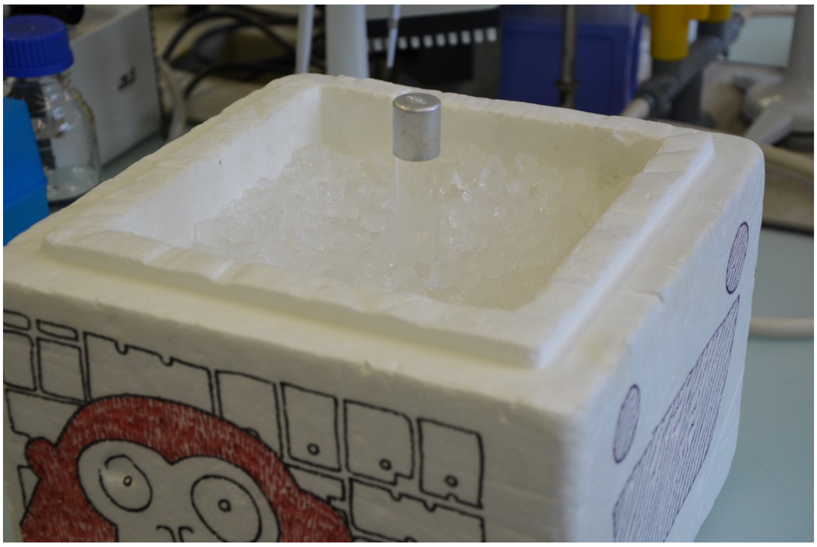

- Quickly plunge the tube in ice and let it stand for 10 minutes

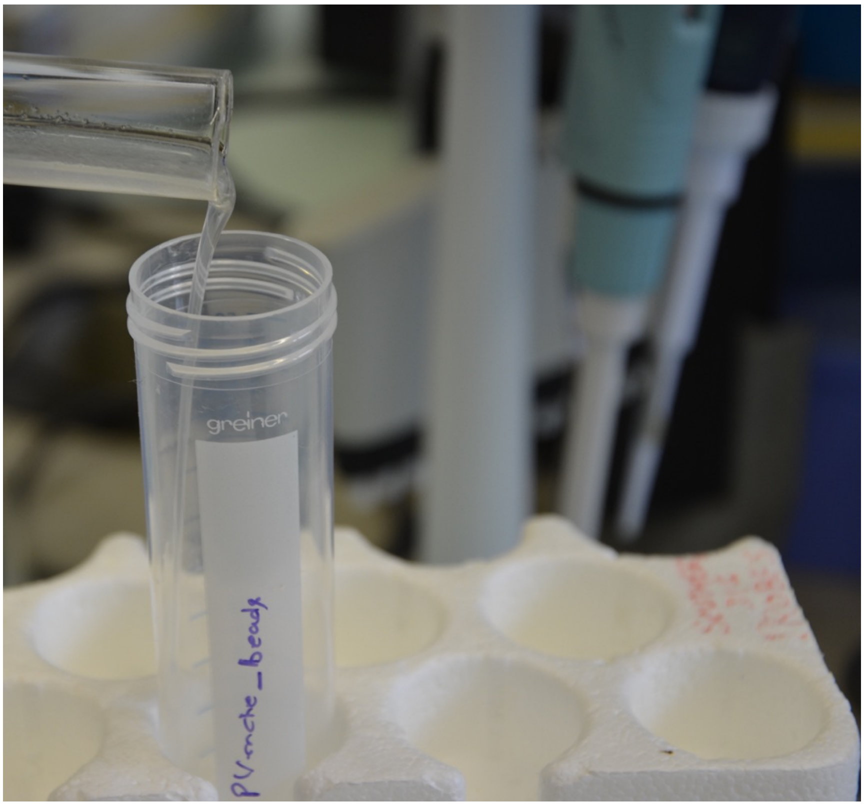

- Transfer the content from the tube to a 50 ml falcon tube
- Centrifuge @2000 rpm for 10 minutes (at room temperature)

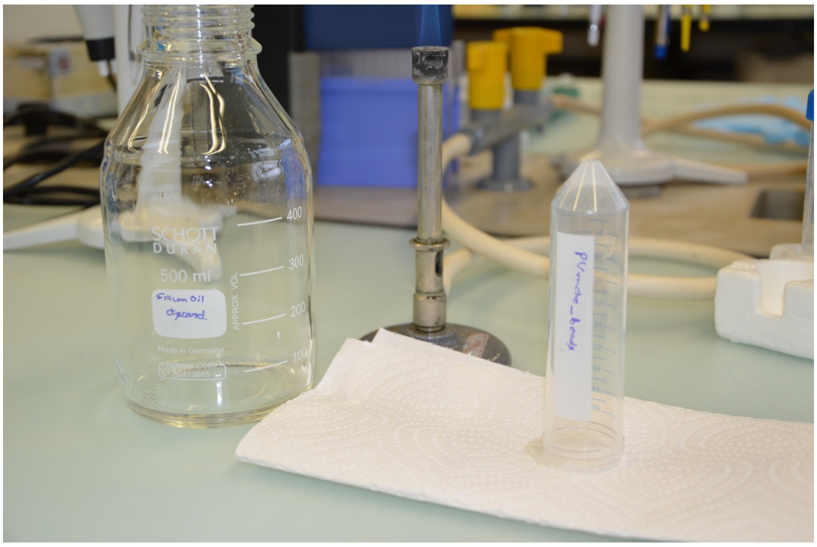

- Decant the oil slowly
- Allow the tube to stand upside down for 3 minutes (max)
- Add 15 ml 1xPBS and centrifuge @2000rpm for 10 minutes (RT) to settle the beads

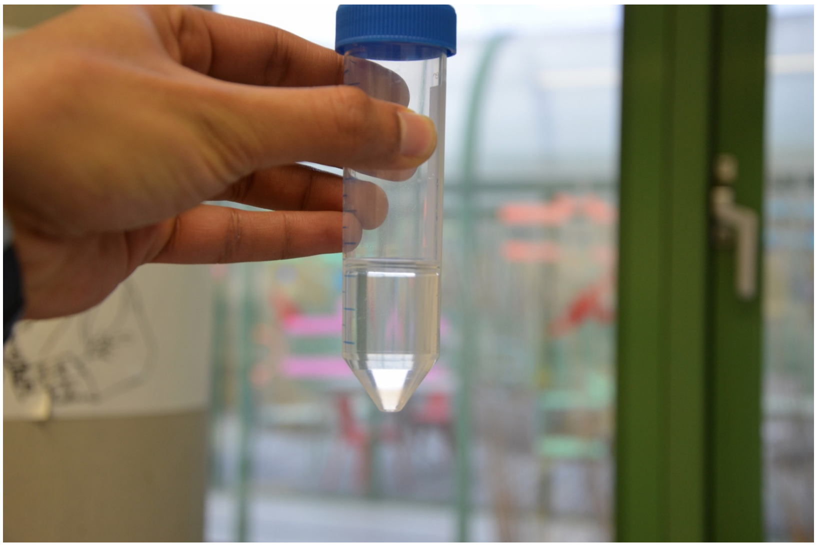

- Remove the liquid (1 ml each time)
- Keep 1 volume of liquid above the beads
- Add 4 ml PBS, centrifuge as before, remove liquid and keep one volume of the liquid above the beads

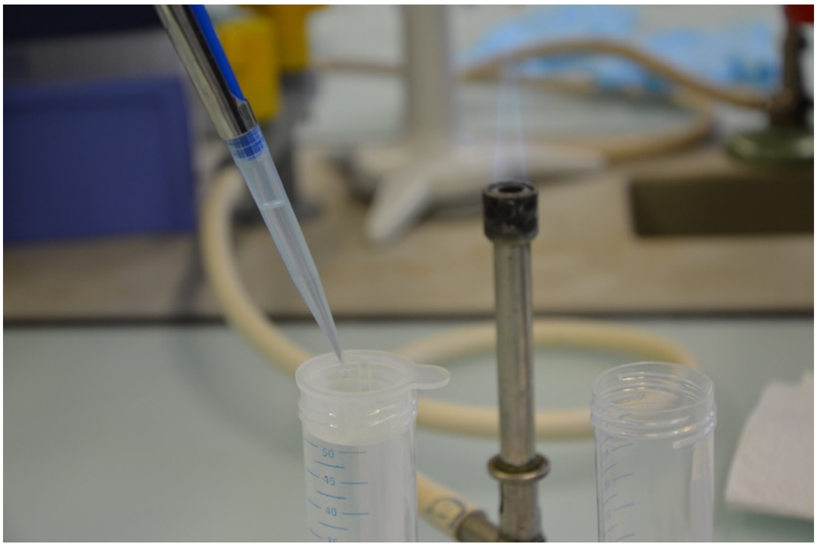

- Filter through a 70 -µm nylon mesh
- take the filtrate

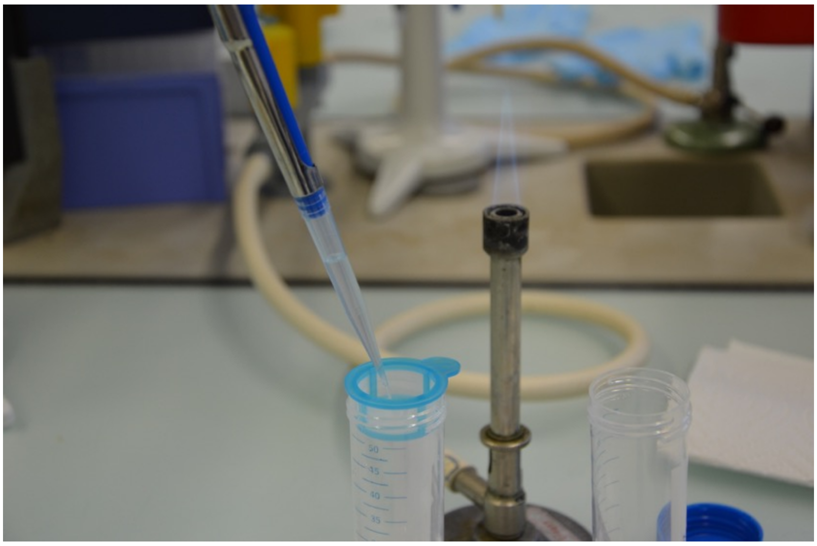

- and pass it through a 40–µm nylon mesh to collect the beads
- Use MOPS to rinse the beads (≥3 ml)
- then turn around to collect them in the desired volume.

Supplementary reference

1. Hadadi, N. & van der Meer, J. R. Soil Community Growth and Diversity Simulations under Low and High Connectivity Conditions (Version MATLAB 2016a) (Zenodo, 2021). DOI: http://doi.org/10.5281/zenodo.4568347
