# Supplementary material for: Environmental connectivity controls diversity in soil microbial communities
Source: Commun Biol. 2021 Apr 22;4:492. doi: 10.1038/s42003-021-02023-2 (PMC8062517; doi:10.1038/s42003-021-02023-2)
Supplement: Supplementary file 3 — Description of Additional Supplementary Files [file 42003_2021_2023_MOESM3_ESM.pdf]

Description of Additional Supplementary Files

File name: Supplementary Data 1

Description: Source data to Figure 2.

File name: Supplementary Data 2

Description: Source data to Figure 3.

File name: Supplementary Data 3

Description: Source data to Figure 4.

File name: Supplementary Data 4

Description: Source data to Figure 5.

File name: Supplementary Data 5

Description: Source data to Figure 6.
