# Supplementary material for: Environmental connectivity controls diversity in soil microbial communities
Source: Commun Biol. 2021 Apr 22;4:492. doi: 10.1038/s42003-021-02023-2 (PMC8062517; doi:10.1038/s42003-021-02023-2)
Supplement: Supplementary file 9 — Reporting Summary [file 42003_2021_2023_MOESM9_ESM.pdf]

nature research

Corresponding author(s): Jan van der Meer
Last updated by author(s): 03.03.2021

Reporting Summary

Nature Research wishes to improve the reproducibility of the work that we publish. This form provides structure for consistency and transparency in reporting. For further information on Nature Research policies, see our Editorial Policies and the Editorial Policy Checklist.

Statistics

For all statistical analyses, confirm that the following items are present in the figure legend, table legend, main text, or Methods section.

n/a	Confirmed	
☐	☒	The exact sample size (n) for each experimental group/condition, given as a discrete number and unit of measurement
☐	☒	A statement on whether measurements were taken from distinct samples or whether the same sample was measured repeatedly
☐	☒	The statistical test(s) used AND whether they are one- or two-sided<br>*Only common tests should be described solely by name; describe more complex techniques in the Methods section.*
☒	☐	A description of all covariates tested
☐	☒	A description of any assumptions or corrections, such as tests of normality and adjustment for multiple comparisons
☐	☒	A full description of the statistical parameters including central tendency (e.g. means) or other basic estimates (e.g. regression coefficient) AND variation (e.g. standard deviation) or associated estimates of uncertainty (e.g. confidence intervals)
☐	☒	For null hypothesis testing, the test statistic (e.g. F, t, r) with confidence intervals, effect sizes, degrees of freedom and P value noted<br>*Give P values as exact values whenever suitable.*
☒	☐	For Bayesian analysis, information on the choice of priors and Markov chain Monte Carlo settings
☐	☒	For hierarchical and complex designs, identification of the appropriate level for tests and full reporting of outcomes
☒	☐	Estimates of effect sizes (e.g. Cohen's d, Pearson's r), indicating how they were calculated

Our web collection on statistics for biologists contains articles on many of the points above.

Software and code

Policy information about availability of computer code

Data collection	Illumina MiSeq short read sequencing for community diversity. Bead imaging with Leica AF6000 LX system. Novocyte flow cytometry instrument software (ACEA).
Data analysis	Sequence reads were processed using the QIIME2 pipeline and attributed to OTUs using the SILVA taxonomic reference gene database. Images were processed using a custom MatLab script (vs 2016a/2017a), provided as supplementary script from Zenodo (http://doi.org/10.5281/zenodo.4568347). Community models were scripted in MatLab (vs 2016a). Script is annexed in Supplementary information and is available for download from Zenodo.

For manuscripts utilizing custom algorithms or software that are central to the research but not yet described in published literature, software must be made available to editors and reviewers. We strongly encourage code deposition in a community repository (e.g. GitHub). See the Nature Research guidelines for submitting code & software for further information.

Data

Policy information about availability of data

All manuscripts must include a data availability statement. This statement should provide the following information, where applicable:

- Accession codes, unique identifiers, or web links for publicly available datasets
- A list of figures that have associated raw data
- A description of any restrictions on data availability

All raw sequences of the community studies have been deposited to the NCBI short read archive. Bioproject number is included in the M&M section. Raw bead images are available from the corresponding author upon request. All data presented in the figures are given as separate Excel source files.

Field-specific reporting

Please select the one below that is the best fit for your research. If you are not sure, read the appropriate sections before making your selection.

☐ Life sciences ☐ Behavioural & social sciences ☒ Ecological, evolutionary & environmental sciences

For a reference copy of the document with all sections, see nature.com/documents/nr-reporting-summary-flat.pdf

Ecological, evolutionary & environmental sciences study design

All studies must disclose on these points even when the disclosure is negative.

Study description	We contrast microbial community growth and the development of community diversity in fully suspended culture versus encapsulated in beads. Each experiment was started with freshly harvested soil community from the same (outside) site, and carried out in biological triplicates. We tested two substrate regimes (soil extract and mixed-carbon), which each were repeated three times independently (at different times of the year).
Research sample	Research samples consist of native soil microbial communities. This is a relevant sample, because soil microbial communities are among the most diverse known; and would experience the concept of 'environmental connectivity' that we aim to study here.
Sampling strategy	This is a combined laboratory experimental study, so there is no specific sampling strategy. The source material for the soil microbial community was sampled at the same geographical spot, and fresh for each experiment so as not to introduce any bias due to difficulties in preserving microbial species in the lab.<br>The experimental sampling was then based on preliminary and preparatory studies to calibrate the amount of substrate needed to observe growth of the various microorganisms. This led to our design of sampling at t=0 (time of start and mixing all components), t = 6 h, t =24, 48 and 72 h (after which growth was complete and the substrate was depleted).
Data collection	We collected two types of data: (i) community and subpopulation growth, and (ii) microbial diversity changes. Community and subpopulation growth in suspended growth was derived from flow cytometry analysis that counts the total number of cells in solution. Growth in beads was estimated from image analysis on microbial microcolonies that were stained with fluorescent dyes for improved differentiation.<br>For diversity analysis, we isolated DNA from cells in beads or cells in solution. This DNA was then used to amplify the V3-V4 region of the 16S rRNA gene, which was further processed for high throughput sequencing. Cleaned and mapped reads to known taxa were then used to infer diversity changes.
Timing and spatial scale	See above for sampling strategy. We did not further introduce a spatial scale.
Data exclusions	No data were excluded from the analysis. Certain experimental repetitions were excluded when they did not show any growth of cells in beads, or too much growth and beads did not remain intact.
Reproducibility	Bead experiments were repeated three times independently at different times of the year and with two different substrates, in order to get an idea of reproducibility of our findings. The community diversity analysis by sequencing was then limited to three of such experiments, each conducted in triplicates. Liquid suspended growth was conducted in biological quadruplicates, but only with a single source material of fresh soil microbial community.
Randomization	Samples were taken from well-mixed liquid suspensions of cells or cells-in-beads. Each biological replicate was sampled once. Every sample was measured in 10-15 technical replicates (for image analysis) or two technical replicates (for flow cytometry).
Blinding	Samples were not further blinded. We think this is irrelevant for our study design.

Did the study involve field work? ☐ Yes ☒ No

Reporting for specific materials, systems and methods

We require information from authors about some types of materials, experimental systems and methods used in many studies. Here, indicate whether each material, system or method listed is relevant to your study. If you are not sure if a list item applies to your research, read the appropriate section before selecting a response.

Materials & experimental systems

n/a	Involved in the study
☒	☐ Antibodies
☒	☐ Eukaryotic cell lines
☒	☐ Palaeontology and archaeology
☒	☐ Animals and other organisms
☒	☐ Human research participants
☒	☐ Clinical data
☒	☐ Dual use research of concern

Methods

n/a	Involved in the study
☒	☐ ChIP-seq
☐	☒ Flow cytometry
☒	☐ MRI-based neuroimaging

Flow Cytometry

Plots

Confirm that:

☐ The axis labels state the marker and fluorochrome used (e.g. CD4-FITC).

☐ The axis scales are clearly visible. Include numbers along axes only for bottom left plot of group (a 'group' is an analysis of identical markers).

☐ All plots are contour plots with outliers or pseudocolor plots.

☐ A numerical value for number of cells or percentage (with statistics) is provided.

Methodology

Sample preparation	We counted bacterial live and dead cells in suspended cell samples from the incubations detailed above. Cell samples from the mixed liquid suspension growth experiments were diluted to approximately 10e6 per ml, subsampled to aliquots of 100 µl, which were mixed with 100 µl of 8 g l–1 sodium azide in phosphate buffered saline, and incubated for 1 h at 4°C to arrest cell respiration and growth. Cell suspensions were diluted 100 times in salt medium and stained in 200 µl aliquots with 2 µl of diluted SYBR Green I solution (1:100 in DMSO; Molecular Probes) in the dark for 30 minutes at room temperature. In some experiments, cells were additionally stained with 2 µl propidium iodide solution (10 µg ml–1, Molecular Probes).
Instrument	Novocyte flow cytometer with absolute volumetric cell counting (ACEA Biosciences, USA). ACEA NovoCyte 2060 incl. NovoSampler and ACEA NovoCyte 3000 incl. NovoSampler.
Software	NovoExpress software from both machines was used for the primary collection of cell data and gating, and reporting of absolute volumetric counts. This was then exported as .csv files and further treated by Excel or MatLab.
Cell population abundance	Bacterial cells were thresholded above a forward scatter signal (FSC-H) of 20 and further gated for propidium iodide-staining (excited at 535 nm and its fluorescence was collected at 617 ± 30 nm) and for SYBR Green I (excitation 488 nm, 530±30 nm band-pass filter; channel voltage at 441 V) above values of 1000.
Gating strategy	We counted particles as bacterial cells when their SYBR Green I fluorescence in the FITC-H channel was above 1000, and their forward scatter (FSC-H) was above 20. Cells were considered PI-positive, if their fluorescence value in the PI-channel was above 1e3.5 and SYBR Green I fluorescence was below 1e3.5.

☒ Tick this box to confirm that a figure exemplifying the gating strategy is provided in the Supplementary Information.
